# CRAC channel opening is determined by a series of Orai1 gating checkpoints in the transmembrane and cytosolic regions

**DOI:** 10.1074/jbc.RA120.015548

**Published:** 2020-12-29

**Authors:** Adéla Tiffner, Romana Schober, Carmen Höglinger, Daniel Bonhenry, Saurabh Pandey, Victoria Lunz, Matthias Sallinger, Irene Frischauf, Marc Fahrner, Sonja Lindinger, Lena Maltan, Sascha Berlansky, Michael Stadlbauer, Rainer Schindl, Rudiger Ettrich, Christoph Romanin, Isabella Derler

**Affiliations:** 1 Institute of Biophysics, JKU Life Science Center, Johannes Kepler University Linz, Linz, Austria; 2 Center for Nanobiology and Structural Biology, Institute of Microbiology, Czech Academy of Sciences, Nove Hrady, Czechia; 3 Gottfried Schatz Research Center, Medical University of Graz, Graz, Austria; 4 College of Biomedical Sciences, Larkin University, Miami, Florida, USA; 5 Faculty of Mathematics and Physics, Charles University, Prague, Czechia; 6 Department of Cellular Biology & Pharmacology, Herbert Wertheim College of Medicine, Florida International University, Miami, Florida, USA

**Keywords:** CRAC channel, STIM1, Orai1, Signal propagation, Gating, Gating checkpoints, Opening-permissive conformation, Electrophysiology, AND-gate, CETR, cytosolic extended transmembrane region, CRAC, Ca^2+^ release activated Ca^2+^, GoF, gain of function, LoF, loss of function, MTR, middle transmembrane region, STIM1, stromal interaction molecule 1, TM, transmembrane domain, V_rev_, reversal potential

## Abstract

The initial activation step in the gating of ubiquitously expressed Orai1 calcium (Ca^2+^) ion channels represents the activation of the Ca^2+^-sensor protein STIM1 upon Ca^2+^ store depletion of the endoplasmic reticulum. Previous studies using constitutively active Orai1 mutants gave rise to, but did not directly test, the hypothesis that STIM1-mediated Orai1 pore opening is accompanied by a global conformational change of all Orai transmembrane domain (TM) helices within the channel complex. We prove that a local conformational change spreads omnidirectionally within the Orai1 complex. Our results demonstrate that these locally induced global, opening-permissive TM motions are indispensable for pore opening and require clearance of a series of Orai1 gating checkpoints. We discovered these gating checkpoints in the middle and cytosolic extended TM domain regions. Our findings are based on a library of double point mutants that contain each one loss-of-function with one gain-of-function point mutation in a series of possible combinations. We demonstrated that an array of loss-of-function mutations are dominant over most gain-of-function mutations within the same as well as of an adjacent Orai subunit. We further identified inter- and intramolecular salt-bridge interactions of Orai subunits as a core element of an opening-permissive Orai channel architecture. Collectively, clearance and synergistic action of all these gating checkpoints are required to allow STIM1 coupling and Orai1 pore opening. Our results unravel novel insights in the preconditions of the unique fingerprint of CRAC channel activation, provide a valuable source for future structural resolutions, and help to understand the molecular basis of disease-causing mutations.

Calcium (Ca^2+^) ions are essential second messengers in the cell that control a variety of processes in immune cells and other cell types (1, 2, 3). One main Ca^2+^ entry pathway into the cell represents the Ca^2+^ release-activated Ca^2+^ (CRAC) channel, which is activated in response to intracellular Ca^2+^ store depletion (4, 5).

CRAC channels are composed of two molecular key players, the Orai proteins (Orai1–Orai3), forming the Ca^2+^ ion channel in the plasma membrane, and the stromal interaction molecule 1 (STIM1), a Ca^2+^ sensor in the membrane of the endoplasmic reticulum (5, 6, 7, 8, 9, 10, 11, 12, 13, 14, 15, 16, 17). Upon Ca^2+^ store depletion of the endoplasmic reticulum, a signaling cascade is initiated finally leading to the coupling of STIM1 oligomers to Orai channels (16, 18, 19, 20, 21, 22, 23, 24, 25, 26), which enables Ca^2+^ ions to enter the cell (27, 28). The stoichiometry of the active STIM1–Orai1 complex is still under debate. At present, three different models exist, which include a unimolecular and a bimolecular model, both with a STIM1:Orai1 1:1 stoichiometry and a sequential binding model with a 2:1 ratio. Although the unimolecular model suggests that each subunit of a STIM1 dimer couples to an Orai1 subunit of two distinct Orai1 complexes (29), the bimolecular model proposes that a STIM1 dimer binds to two adjacent Orai1 subunits within one channel (30). The sequential binding model specifies that two STIM1 C termini couple to one Orai1 subunit (31).

Each Orai subunit is composed of four transmembrane domains (TM1–TM4), which are flanked by their N- and C-terminal strands and connected *via* one intracellular (TM2-TM3) and two extracellular (TM1-TM2, TM3-TM4) loops (17, 32, 33). All three cytosolic segments are indispensable for STIM1-mediated Orai activation. The Orai C terminus functions as the main binding site for STIM1 C terminus (20, 27, 34, 35, 36, 37, 38). Concerning the N terminus and the loop2, it is not yet clear whether they form weak STIM1 binding sites or affect STIM1 binding allosterically (37, 39). At present, there is evidence that the loop2 region is critical in STIM1-mediated Orai1 gating (40) and controls the interplay with the Orai N terminus to adjust a permissive Orai channel conformation (39). Furthermore, the Orai N terminus communicates with STIM1 to govern the authentic CRAC channel hallmarks (41), including high Ca^2+^ selectivity, fast Ca^2+^ dependent inactivation, and enhancement in currents in a divalent-free Na^+^- versus a Ca^2+^-containing solution. There are currently two hypotheses how pore opening is generated. Either STIM1 coupling to Orai1 C terminus is sufficient to trigger pore opening or the direct interaction of the C termini of STIM1 and Orai1 is followed by a coupling of STIM1 to the loop2 and/or N terminus to fully establish the Orai1 activation authentic for CRAC channels.

The four currently published crystal and two cryo-EM structures of *Drosophila melanogaster* Orai (dOrai) determine that Orai ion channels form hexameric assemblies (42, 43, 44, 45). The Ca^2+^ pore is formed by six TM1 domains centered in the middle of the channel complex. They are surrounded by the TM2 and TM3 regions in a second ring and by TM4 in a third ring. Among the four dOrai structures, two are representative of the closed state (dOrai, dOrai K163W) (42). The other published structures are assumed to constitute open channel variants, as they contain one of the two well-known constitutively activating point mutations H206A (in human Orai1 H134A) (44, 45) and P288L (in human Orai1 P245L) (43), respectively. Moreover, these structures unveil unique features of the cytosolic strands. The last 20 amino acids of the Orai1 N terminus form a helical extension of TM1, thus contributing to the cytosolic part of the pore. The C termini form helical regions connected to TM4. A comparison of the structures reveals consistent results on potential conformational changes upon Orai activation within the inner part of the channel complex including TM1–TM3. Structural resolutions indicate that the basic region in TM1 expands by approximately 10 Å from the closed to the open state. On the contrary, the structural changes upon pore opening of TM4 and the C terminus at the outmost side of the channel complex are still a matter of debate, owing to discrepancies in the structural resolutions (42, 43, 44).

The initial signal for Orai pore opening represents STIM1 coupling to the Orai1 C terminus (36, 46). This might lead to conformational changes of the C termini in the Orai complex, which are further transmitted to the pore region. Site-directed and cysteine scanning mutagenesis studies exhibited that not only amino acids in the pore-lining TM1 but overall 15 positions within all TM domains contribute to the maintenance of the closed state (36, 39, 41, 47, 48, 49, 50, 51, 52, 53, 54, 55), as their single point mutations can lead to constitutive activity. Critical regions for Orai1 pore opening represent the hinge connecting TM4 and the C terminus, the TM2/3 ring, a hydrophobic cluster at the TM1-TM2/3 interface, a serine ridge at the TM1-TM2/3 interface, the H134 residue in TM2, sulfur-aromatic interactions of TM1 and TM3 (56), and the N terminus (41, 57, 58). Pore opening is assumed to be facilitated by a small rotation of the hydrophobic region in TM1 (45, 58) as well as by the presence of basic residues at the inner pore (51, 59). It is worth noting that the recent dOrai H206A cryo-EM structure resolved no helix rotation of the pore-lining TM1 domain (45). Finally, signal propagation from the outmost side to the pore in the center of the channel complex has been suggested to involve interdependent conformational changes of all TM helices (57). A recent computational model indicates a “twist-to-open” Orai gating mechanism, which involves a collective counterclockwise rotation of the extracellular part and two independent types of motions of the intracellular side of the channel complex (60).

In our study, we provide solid proof for this interdependent communication of the Orai TM helices using a library of Orai double point mutants systematically combining one gain (GoF)- and one loss-of-function (LoF) single point mutation (Table 1). We hereby present a map of critical checkpoints that are all required to adopt an opening-permissive conformation to guarantee pore opening. Our characterization of a variety of LoF mutations distilled certain basic and acidic residues in the cytosolic portions of TM domains in wildtype Orai1, which form triangular salt bridge interactions and possess a pivotal role in pore opening and functional STIM1 coupling.

**Table 1:** Summary of Orai1-GoF-LoF double mutants with either the LoF or GoF mutation acting in a dominant manner over the GoF or LoF mutation, respectively

This table differentiates between inactive (LoF dominant over GoF) and active (GoF dominant over LoF) Orai1-GoF-LoF double mutants, which emphasizes whether either the LoF or the GoF mutation has a dominant effect. The double mutants are grouped according to the location of the LoF and GoF mutation either in the MTR or CETR. In addition, the double mutants are sorted according to the location of the LoF and GoF mutations in one of the TM domains. Inactive double mutants with the LoF mutation closer to the pore are highlighted in orange, the GoF mutations closer to the pore are labeled blue, and the GoF and LoF mutations in the same TM domain are transparent. Active double mutants containing V102A are labeled in yellow. Other active double mutants are shown in green.

CETR, cytosolic extended transmembrane region; GoF, gain of function; LoF, loss of function; MTR, middle transmembrane region; TM, transmembrane domain.

## Results

### Mutation of several residues located in the middle of TM2, TM3, and TM4 of Orai1 channels can lead either to GoF or LoF mutants

**Figure 1:**
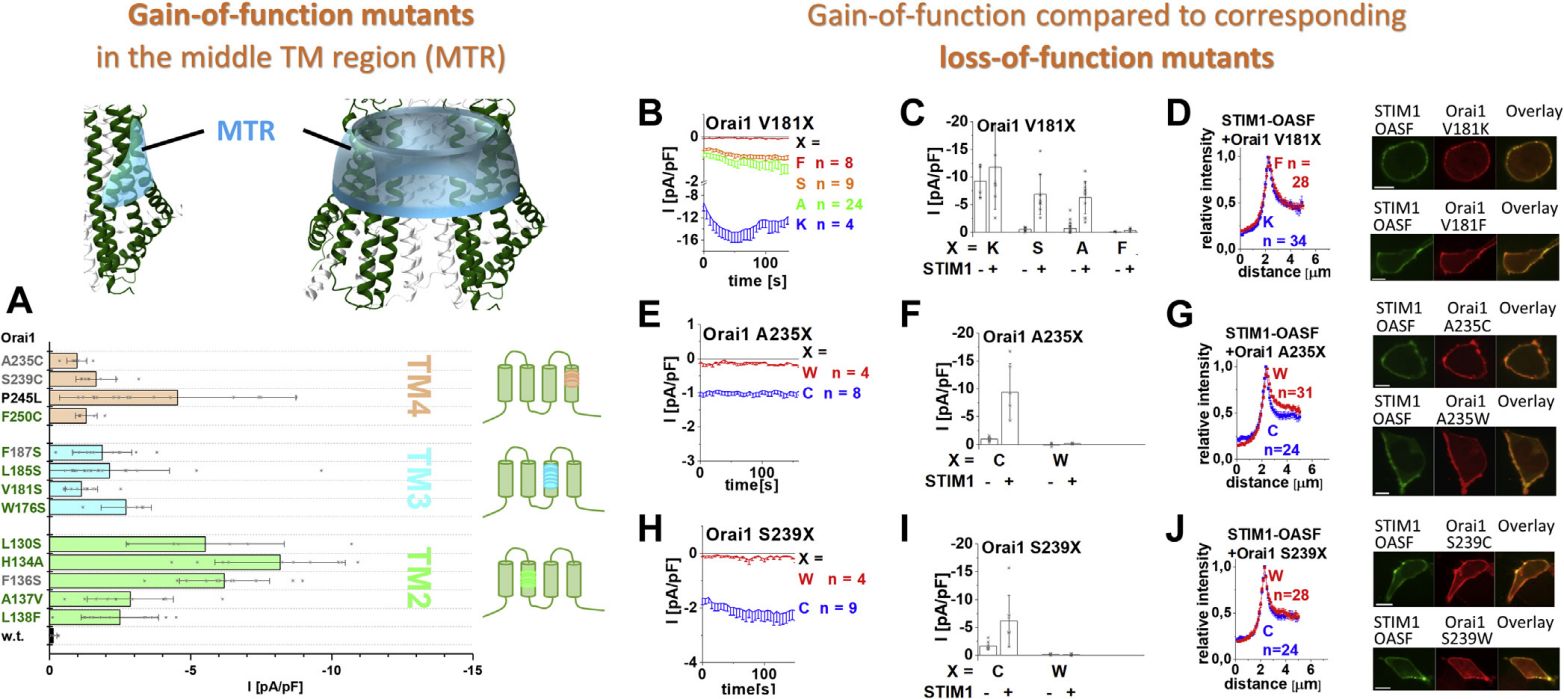
**A screen on Orai1 TM domains revealed several GoF mutants, exhibiting constitutive activity independent of store depletion and STIM1, and some corresponding LoF mutant.** Scheme representing the location of the MTR (*cyan*) within one Orai subunit (*left*) and the whole channel complex (*right*). *A*, electrophysiological screen of residues in TM2 (*green*), TM3 (*cyan*), and TM4 (*orange*) *via* single point mutations, with the focus on those located in close proximity (2–4 Å) within one or between two adjacent Orai subunits (Tables 2 and 3; include distances estimated *via* Pymol in the hOrai1 model (51, 63) based on the X-ray structure of the closed dOrai Protein Data Bank ID: 4HKR) revealed several Orai1 mutants that are constitutively active independent of store depletion and STIM1. The block diagram shows the current densities of the respective constitutively active point mutants containing the amino acid substitution in TM2 (*green*), TM3 (*blue*), or TM4 (*orange*) (n = 6–19 cells; values are mean ± SD). Current densities of constitutive GoF mutants are significantly different from currents obtained with Orai1 wildtype (Welch-ANOVA F(13, 43.68) = 36.43, *p* < 0.001; Games–Howell post hoc test revealed a significant difference between the GoF mutants and the Orai1 wildtype [*p* < 0.05]). The color code used for the mutations refers to their close proximity to a residue within one subunit (*green*) or an adjacent subunit (*gray*). P245L is shown in *black* as it causes constitutive activity, but is not located in close proximity to residues of adjacent TM domains. *B*, *E*, *H*, time courses represent current densities after whole-cell break-in of novel Orai mutants containing the LoF mutation V181F in comparison with the GoF mutations V181S, V181A, V181K (*B*), the LoF mutation A235W in comparison with the GoF mutation A235C (*E*), and the LoF mutation S239W in comparison with the GoF mutation S239C (*H*) in the absence of STIM1. *C*, *F*, *I*, block diagram with current densities of mutants from (*B*), (*E*), and (*H*) in the absence (t = 0 s) compared with the presence (maximum current density) of STIM1. The current densities differed statistically significantly for the different Orai1 variants (Welch-ANOVA F(7, 19.96) = 20.29, *p* < 0.001 [*C*], F(3, 7.39) = 17.95, *p* < 0,001 [*F*], F(3, 11.18) = 15.79, *p* < 0.001 [*I*]) The Games–Howell post hoc test revealed a significant difference between the GoF mutants and the corresponding LoF mutants (*p* < 0.05). *D*, *G*, *J*, intensity plots of STIM1–OASF coexpressed with Orai1 V181K compared with Orai1 V181F (*D*), with Orai1 A235C compared with Orai1 A235W (*G*), and with Orai1 S239C compared with Orai1 S239W (*J*) (at 4 μm, Mann–Whitney test *p* > 0.05 for each pair in [*D*], [*G*], and [*J*]). Corresponding image series depict YFP-Orai1 mutants shown in (*D*), (*G*), and (*I*), CFP-OASF and overlay (the scale bar represents 10 μm). GoF, gain of function; LoF, loss of function; MTR, middle transmembrane region; TM, transmembrane domain.

Several single point mutants of Orai1, containing amino acid substitutions, in distinct TM regions, have already been reported to display constitutive activity (37, 41, 46, 48, 50, 51, 52, 55, 57, 58). This led to the hypothesis that Orai channel opening is controlled by several critical sites within all four TM regions (40, 41, 51, 57, 61, 62). We screened residues in TM2/3 and 4 *via* single point mutations, with the focus on those located in close proximity (2–4 Å) within one or between two adjacent Orai subunits (Tables 2 and 3; include distances estimated *via* Pymol in the hOrai1 model (51, 63) based on the X-ray structure of the closed dOrai Protein Data Bank ID: 4HKR). We assumed that they are most likely involved in manifesting a stable closed or open conformation (57) by forming noncovalent interactions with each other. As the mutation of those sites should not disturb the TM helix itself, most residues, with a few exceptions (*e.g.*, V181K, S239W), were exchanged by ones with a preference for helix formation such as serine or alanine, but not by beta-branched residues, which might disrupt the helix (64). We discovered, among the residues located in close proximity of adjacent TM domains (Tables 2 and 3), an overall number of 13 positions in the Orai1 helices TM2, TM3, and TM4 lead to constitutive activity upon single point mutation (Figs. 1*A* and S1, *A*–*C*). Our screen is largely in line with the cysteine screen of Yeung *et al*. (57) except for some variations. They identified overall 11 positions with TM2, TM3, and TM4 that maintain the closed state (57). A comparison of the cysteine screen with our screen on residues in close proximity revealed that we discovered five novel positions involved in the maintenance of the closed state (L130, F136, L138, V181, L185). Among the novel gain-of-function (GoF) single point mutations within Orai1, we elucidated the following six: in TM2 L130S, F136S, in TM3 W176S, V181S/K, F187S (Figs. 1, *A–C*, S2I, *A–J*, and S2II, *A–J*), in addition to the reported H134A, A137V (51), L138F (52), W176C (53), V181A, L185A (39, 41), and diverse cysteine substitutions (57). The constitutive TM4 mutants Orai1 A235C, S239C, F250C (57), and P245L (46) behaved in an identical manner to the recently reported ones (Figs. 1, *A*, *E*, *F*, *H*, and *I*, S2I, *K–R*, and S2II, *K–R*). In general, except for the positions A137 and L138, a substitution of a strongly hydrophobic residue to a serine, alanine, or cysteine destabilizes the closed Orai1 channel conformation and induces constitutive activity.

**Table 2:** Residues that are located in close proximity within one Orai subunit

Color code: Pairs of residues located in close proximity between TM1-TM2 (orange), TM1-TM3 (gray), TM2-TM3 (blue), and TM3-TM4 (green) and corresponding distance of the respective side chains.

TM, transmembrane domain.

**Table 3:** Residues that are located in close proximity between two adjacent Orai subunits

Color code: Pairs of residues located in close proximity between TM1^2^-TM2^1^ (dark red), TM2^1^-TM3^2^ (light blue), and TM2^1^-TM4^2^ (purple) and corresponding distance of the respective side chains; ^1^ and ^2^ indicate adjacent Orai1 subunits.

TM, transmembrane domain.

^a^Our MD simulations reveal that K85–E149 comes in more than 50% of the detected distances closer than 4 Å.

In contrast, the exchange of a few of those residues by ones with strongly distinct properties of size and/or hydrophobicity can lead to loss-of-function (LoF) mutants (Figs. S2I, *A–R* and S2II, *A–R*). This fact has already been reported for Orai1 H134A and Orai1 L138F, which are constitutively active, whereas Orai1 H134W and L138A remain inactive also in the presence of STIM1 (51). Moreover, we discovered loss of function of Orai1 V181F in TM3, Orai1 A235W and S239W in TM4, both, in the absence (Figs. 1, *B*, *C*, *E*, *F*, *H*, and *I*, S2I, and S2II, *E*, *K*, and *M*) as well as the presence of STIM1 (Figs. 1, *C*, *F*, and *I*, S2I, and S2II, *F*, *L*, and *N*). It seems that especially large, bulky residues can lead to a loss of function, however, owing to exceptions (L138F, Figs. 1*A* and S2II) no clear rules can be postulated. There is no obvious dependence of gain or loss of function in terms of hydrophobicity (Kyte and Doolittle scale [65]) of the introduced residue in line with previous findings (Fig. S2I) (57). At some other positions, such as L185, F187, and P245, any amino acid substitution leaves the Orai1 channel in a functional state, either constitutively or activatable by STIM1 (Figs. S2I and S2II, *G–J*, *O*, and *P*) (46). Also, the substitution of L130 and F136 by the small polar serine or the bulky, hydrophobic tryptophan retained both store-operated activity (Figs. S2I and S2II, *A–D*).

To investigate whether loss of function of the above-mentioned mutants is partially a result of impaired STIM1 coupling, we investigated the intensities of the Orai1-activating STIM1 C-terminal fragment, OASF (aa 233–474), in the presence of diverse LoF compared with GoF mutants. However, both GoF and the corresponding LoF point mutants revealed comparable colocalization intensities with OASF (Fig. 1, *D*, *G*, and *J*) compatible with similar affinities among the Orai1 mutants.

In summary, our screening on nearby residues from adjacent TM domains within and between Orai1 subunits led to the discovery of in total 13 positions in Orai1 TM2/3/4 that contribute to the establishment of the closed state of the Orai1 channel. They can become constitutively active upon single point mutation to certain amino acids independent of the presence of STIM1. Some of them are additionally involved in the maintenance of an opening-permissive pore geometry or signal propagation, since distinct amino acid substitutions at those positions can lead to loss of function, without interfering with the coupling to STIM1-OASF. Overall, all these positions are concentrated in a conical ring spanning across the middle plane of the TM2/3/4 domains and a segment of TM4 closer to the extracellular side, which we call middle transmembrane region (MTR) (Fig. 1, scheme of subunit and channel).

### MTR-LoF Orai1 point mutations act dominant over MTR-GoF mutations independent of their location relative to each other

**Figure 2:**
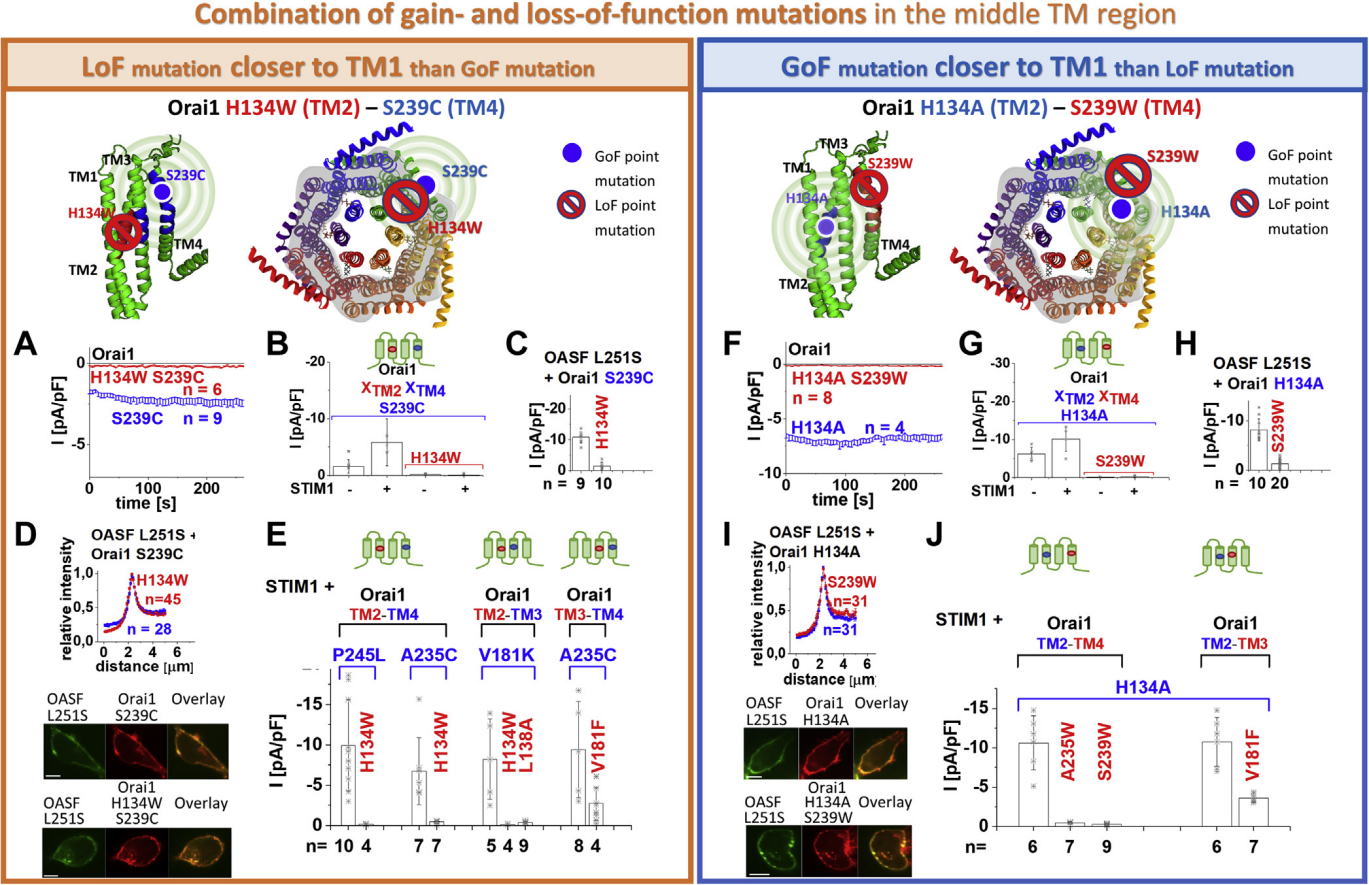
**Constitutive currents of MTR-GoF mutants are inhibited by MTR-LoF mutations in a dominant manner independent of their location relative to each other.** Schemes representing the location of the investigated residues within a single subunit (*top left*) of Orai1 or the whole channel complex (*top middle*), for either LoF or GoF mutation closer to the pore, respectively. The *red stop sign* represents the position of the LoF mutation, while the *blue circle* shows the position of the GoF mutation. The *spheres* indicate the impact of the GoF mutation on the entire subunit. Special focus was addressed to H134 and S239, owing to their location in TM2, thus, close to the pore, or in TM4, thus, at the periphery of the channel complex, respectively (*A–D*, *F–I*). By combining a GoF and an LoF mutation at the respective positions and investigating their impact on each other, we examined whether interdependent TM domain motions within the entire MTR are necessary for pore opening. To provide a solid foundation for the conclusions obtained with the mutations at H134 and S239, we investigated a diversity of other double mutants combining distinct LoF and GoF mutations at other positions (*E*, *J*). *A*, time courses of current densities after whole-cell break-in of Orai1 S239C compared with Orai1 H134W S239C in the absence of STIM1. Constitutive currents of the MTR-GoF Orai1 S239C mutant are inhibited by the additional introduction of the LoF mutation H134W. *B*, block diagram of whole-cell current densities of Orai1 S239C, Orai1 H134W S239C in the absence (t = 0 s) and the presence (maximum current densities) of STIM1 (n = 6–9 cells; values are mean ± SEM). The current densities differed statistically significantly for the different Orai1 variants (Welch-ANOVA F(3, 11.87) = 12.36, *p* < 0.001. The Games–Howell post hoc test revealed a significant difference between the GoF mutants and the corresponding GoF–LoF double mutants (*p* < 0.05). This holds for paired comparisons both in the absence, both in the presence, or one in the absence and one in the presence of STIM1). *C*, block diagram of whole-cell current densities of Orai1 S239C compared with Orai1 H134W S239C in the presence of STIM1 OASF L251S. Activation of the LoF–GoF Orai1 H134W S239C double mutant *via* STIM1 OASF L251S is significantly reduced compared with that of the GoF Orai1 S239C mutant (Mann–Whitney test *p* < 0.001). *D*, intensity plots of STIM1-OASF-L251S coexpressed with Orai1 S239C compared with Orai1 H134W S239C (at 4 μm, Mann–Whitney test *p* > 0.05). The image series depict YFP-Orai1 S239C or YFP-Orai1 H134W S239C mutants, CFP-OASF-L251S and overlay (the scale bar represents 10 μm). *E*, block diagram of maximum whole-cell current densities of Orai1 GoF and the corresponding Orai1 GoF–LoF mutants. Orai1 P245L compared with Orai1 H134W P245L (Mann–Whitney test *p* < 0.05); Orai1 A235C compared with Orai1 H134W A235C (Mann–Whitney test *p* < 0.05); Orai1 V181K compared with Orai1 H134W V181K and Orai1 L138A V181K (Welch-ANOVA F(2, 7.69) = 13.15; *p* < 0.05; the Games–Howell post hoc test revealed a significant difference for the comparison of the GoF mutant with each GoF–LoF double point mutant [*p* < 0.05]); Orai1 A235C in comparison with Orai1 V181F A235C (Mann–Whitney test *p* < 0.05), all in the presence of STIM1. *F*, time courses of current densities after whole-cell break-in of Orai1 H134A compared with Orai1 H134A S239W in the absence of STIM1. Constitutive currents of the MTR-GoF Orai1 H134A mutant are inhibited by the additional introduction of the LoF mutation S239W. *G*, block diagram of whole-cell current densities of Orai1 H134A, Orai1 H134A S239W in the absence (t = 0 s) and the presence (maximum current densities) of STIM1 (n = 4–9 cells; values are mean ± SD). The current densities differed statistically significantly for the different Orai1 variants (Welch-ANOVA F(3, 8.22) = 32.68, *p* < 0.001. The Games–Howell post hoc test revealed a significant difference between the GoF mutants and the corresponding GoF–LoF double mutants (*p* < 0.05). This holds for paired comparisons both in the absence, both in the presence, or one in the absence and one in the presence of STIM1). *H*, block diagram of whole-cell current densities of Orai1 H134A compared with Orai1 H134A S239W in the presence of STIM1 OASF L251S. Activation of the LoF–GoF Orai1 H134A S239W double mutant *via* STIM1 OASF L251S is significantly reduced compared with that of the GoF Orai1 S239A mutant (Mann–Whitney test *p* < 0.001). *I*, intensity plots of STIM1-OASF-L251S coexpressed with Orai1 H134A compared with Orai1 H134A S239W (at 4 μm, Mann–Whitney test *p* > 0.05). The image series depict YFP-Orai1 H134A or YFP-Orai1 H134A S239W mutants, CFP-OASF-L251S and overlay (the scale bar represents 10 μm). *J*, block diagram of maximum whole-cell currents of Orai1 H134A compared with Orai1 H134A A235W, Orai1 H134A S239W and Orai1 H134A V181F, all coexpressed with STIM1 (Welch-ANOVA F(4, 16.18) = 29.76, *p* < 0.001; Games–Howell post hoc test revealed a significant difference for the comparison of the GoF mutant with each GoF–LoF double point mutant [*p* < 0.05]). GoF, gain of function; LoF, loss of function; MTR, middle transmembrane region; TM, transmembrane domain.

Orai1 channel activation is initiated *via* STIM1 coupling to the Orai1 C terminus (54); however, how this activation signal is transmitted to the pore has so far remained unclear. The huge variety of constitutively active Orai1-TM mutants (Figs. 1, S1, S2I, and S2II) (41, 46, 50, 51, 52, 55, 57, 58) allows one to assume that STIM1 binding successively alters the conformations of TM4 up to TM1 *via* interdependent TM motions, finally leading to pore opening. Here, we exploited the various MTR-GoF and MTR-LoF point mutations to determine to which extent a global conformational change within the entire channel complex is required for pore opening. Therefore, we generated and investigated a pool of double point mutants including one MTR-GoF and one MTR-LoF point mutation in distinct TM domains, one of which is located in a TM region (*e.g.*, TM2) closer to the pore than the other one (*e.g.*, TM4) (Table 1). First, attention was paid to residues H134 in TM2 and S239 in TM4 in order to examine the influence of a LoF and a GoF mutation at these two positions on each other. The reason for the particular focus on H134 and S239 is that one is located near the pore while the other one is at the channel periphery. This makes it possible to study the influence of a LoF and a GoF mutation on each other within the entire MTR. As anticipated, the MTR-LoF point mutation H134W in TM2 combined with the MTR-GoF point mutation S239C in TM4 (Orai1 H134W S239C) led to the loss of function in the absence of STIM1 (Fig. 2, *A* and *B*). Furthermore, the coexpression of STIM1 was unable to restore the function (Fig. 2*B*). Analogously, other combinations with the LoF point mutation closer to the pore, like Orai1 H134W V181K, Orai1 L138A V181K, Orai1 H134W A235C, Orai1 H134W P245L, and Orai1 V181F A235C (Fig. 2*E*), abolished or significantly reduced STIM1-mediated activation. Of interest, the double point mutant including the MTR-GoF point mutation H134A in TM2 combined with the MTR-LoF point mutation S239W in TM4 remained also inactive independent of the presence of STIM1 (Fig. 2, *F* and *G*). Similar combinations with the MTR-GoF point mutation closer to the pore such as Orai1 H134A V181F, Orai1 H134A A235W/S239W showed significantly reduced or eliminated function also in the presence of STIM1 (Fig. 2*J*). Moreover, we tested a double point mutant containing the MTR-GoF and the MTR-LoF point mutation within the same TM domain, specifically TM4. Orai1 A235W P245L also displayed loss of constitutive activity (Fig. S3 *P*), indicating that the diverse MTR-LoF point mutations act in a dominant manner. Control experiments revealed that these nonfunctional double point mutants displayed comparable plasma membrane expression like the associated constitutive single point mutants (Fig. S3, *A*, *F*, *L*, and *V*). In addition to all MTR-GoF mutants, one GoF mutant containing substitutions within the hinge region, Orai1 ANSGA (L261A-V262N-H264G-K265A) is currently known (54). Its constitutive activity can also be overruled by MTR-LoF point mutations (Orai1 H134W ANSGA; Orai1 A235W ANSGA; Fig. S4*N*). Collectively, MTR-LoF mutations overrule the effect of MTR-GoF mutations independent of their position relative to each other, located in either the middle or outer transmembrane ring.

Furthermore, we used the STIM1 C-terminal fragment, STIM1 OASF L251S, already exhibiting an open conformation and thus an enhanced affinity to Orai1 (28), to investigate for potential recovery of the function of the inactive Orai1 double mutants. Both Orai1 H134W S239C and Orai1 H134A S239W revealed in the presence of OASF L251S marginal but significantly reduced currents compared with the constitutively active, single point mutants Orai1 S239C and Orai1 H134A, respectively (Fig. 2, *C* and *H*). Colocalization studies revealed almost unaffected coupling of OASF L251S and STIM1 OASF to the two Orai1 double mutants, Orai1 H134W S239C and Orai1 H134A S239W, compared with the constitutively active single point mutants Orai1 S239C and Orai1 H134A, respectively (Figs. 2, *D* and *I* and S3, *E* and *K*). Also, other double point mutants exhibit affinity for STIM1 OASF to a comparable extent as their corresponding constitutively active point mutants (Fig. S3, *C*–*E*, *K*, and *Q*).

In contrast to the above-mentioned GoF mutations, the constitutively active Orai1 TM1-V102A mutant represents an exception. Its function cannot be overruled by LoF mutations in the MTR, as exemplarily tested for Orai1 V102A H134W (Fig. S4*A*). It shows nonselective currents with a reversal potential (V_rev_) in the range of ∼+ 20 mV, comparable with Orai1 V102A currents (37, 48). The coexpression of STIM1 enhanced V_rev_ to ∼+ 50 mV, similar to STIM1-mediated Orai1 V102A currents (Fig. S4*B*) (37, 48). In contrast to Orai1 V102A, the constitutive activity of other TM1 mutants Orai1 F99M and Orai1 V107M are overruled by LoF mutations, as proven by Orai1 F99M H134W and Orai1 V107M H134W double mutants, which both exhibit loss of function (Fig. S4, *I* and *J*).

In summary, our library of GoF–LoF mutants revealed a dominant role of the LoF over the GoF point mutations within the MTR, except the GoF mutation V102A. This proves, for the first time, that Orai1 pore opening indispensably requires an interdependent communication of the TM helices and a series of Orai1 pore opening-permissive checkpoints in the MTR.

### Novel Orai1 LoF point mutants in TM2, TM3, and TM4

**Figure 3:**
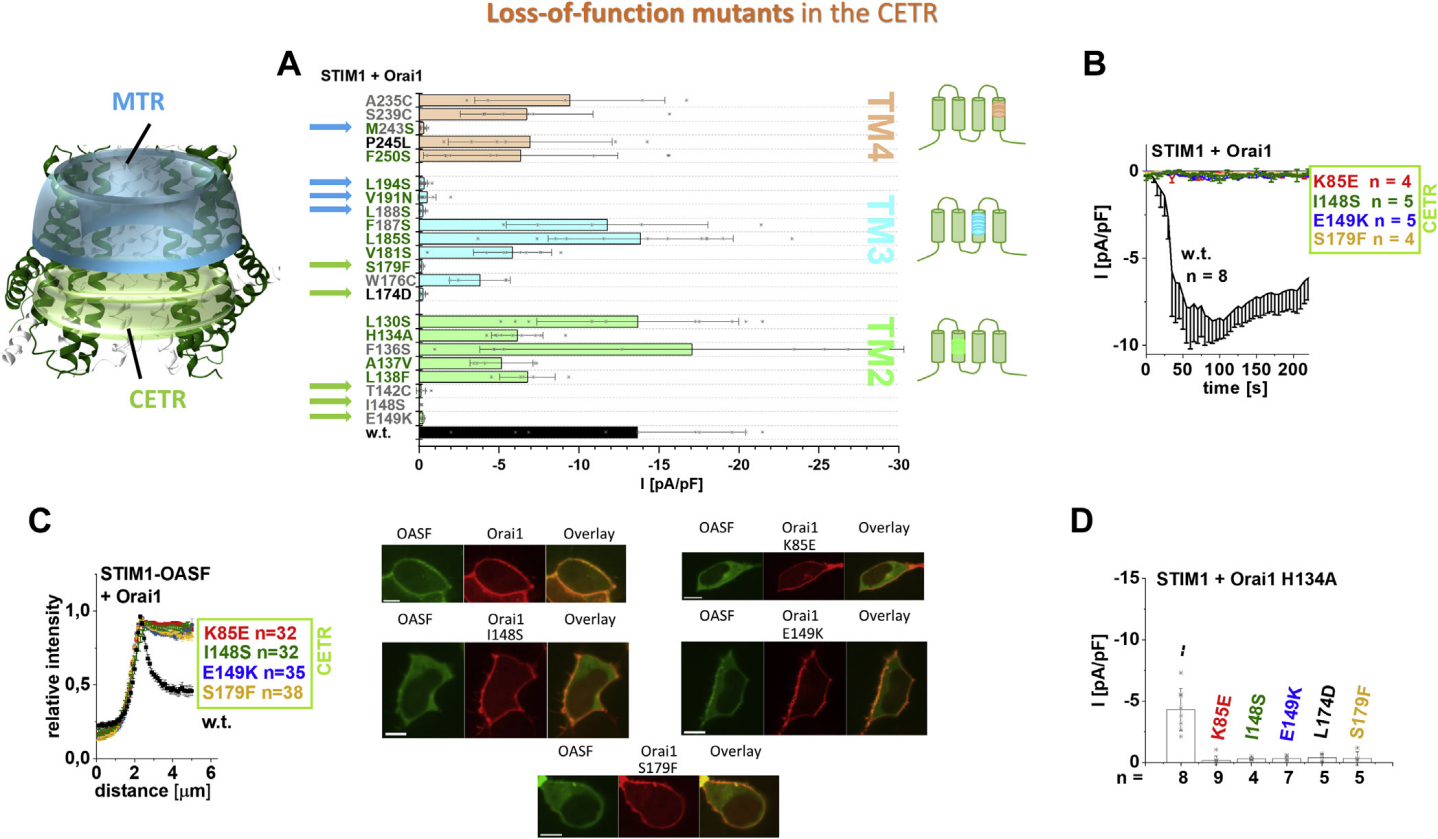
**CETR-LoF mutations impair STIM1 coupling and abolish constitutive activity of Orai1 H134A.** Scheme representing the location of the MTR (*cyan*) and the CETR (*green*) in the whole channel complex. *A*, electrophysiological screen of residues in TM2 (*green*), TM3 (*cyan*), and TM4 (*orange*) *via* single point mutations, with the focus on those located in close proximity (2–4 Å) within one or between two adjacent Orai subunits (Tables 2 and 3; include distances estimated *via* Pymol in the hOrai1 model (51, 63) based on the X-ray structure of the closed dOrai Protein Data Bank ID: 4HKR) revealed several Orai1 mutants that remain inactive in the presence of STIM1. Block diagram showing STIM1-mediated current densities of TM2 (*green*), TM3 (*blue*), and TM4 (*orange*) Orai1 single point mutants (n = 4–12 cells; values are mean ± SD). The current densities differed statistically significantly for the different Orai1 variants (Welch-ANOVA F(22, 65.43) = 11.48, *p* < 0.001). Games–Howell post hoc test revealed a significant difference between the STIM1-mediated Orai1 wildtype and Orai1 T142C, Orai1 I148S, Orai1 E149K in TM2; Orai1 S179F, Orai1 L174D, Orai1 L188S, Orai1 V191N, Orai1 L194S in TM3; and Orai1 M243S currents (*p* < 0.05). Thus, these mutants represent the critical LoF point mutants located either in the MTR (*blue arrows*) or the CETR (*green arrows*). The color code used for the mutants refers to their close proximity to a residue within one subunit (*green*) or an adjacent subunit (*gray*). *B*, time course of STIM1-mediated CETR LoF Orai1 mutant current densities discovered in (*A*). STIM1-mediated current densities of the LoF mutants are significantly different compared with those of the Orai1 wildtype. (Welch-ANOVA F(4, 17.29) = 8.77, *p* < 0.001; Games–Howell post hoc test revealed a significant difference between the STIM1-mediated Orai1 wildtype and each Orai1 LoF mutant shown in (*B*) [*p* < 0.05]). *C*, intensity plots of STIM1-OASF coexpressed with Orai1 mutants shown in (*B*) compared with wildtype Orai1 (at 4 μM, Welch-ANOVA F(3, 72.19) = 73.17, *p* < 0.001; Games–Howell post hoc test revealed significant difference for Orai1 wildtype compared with all CETR mutants, respectively [*p* < 0.05]). Corresponding image series depict respective YFP-Orai1 mutants, CFP-OASF, and overlay (the scale bar represents 10 μm). *D*, block diagram of STIM1-mediated Orai1 double mutant current densities of Orai1 Orai1 K85E H134A, Orai1 H134A I148S, Orai1 H134A E149K, Orai1 H134A L174D, and Orai1 H134A S179F in comparison with Orai1 H134A. Constitutive currents of the MTR-GoF Orai1 H134A mutant are inhibited in a dominant manner *via* the introduction of the LoF mutations K85E, I148S, E149K, L174D, as well as S179F (Welch-ANOVA F(5, 13.29) = 7.55, *p* < 0.005; Games–Howell post hoc test revealed significant difference for GoF-Orai1-H134A compared with GoF-Orai1-H134A mutants containing I148S, E149K, L174D, or S179F). CETR, cytosolic extended transmembrane region; GoF, gain of function; LoF, loss of function; MTR, middle transmembrane region; TM, transmembrane domain.

Our site-directed mutagenesis screen through a series of TM residues located in close proximity to those of adjacent TM domains revealed, besides the above-described MTR-LoF mutations, additional LoF mutations throughout all TM domains (Tables 2 and 3; Fig. S1, *D*–*F*). Newly discovered LoF mutants represent Orai1 T142A/C, I148S (TM2), E149K (TM2), S179F/M/W/T/R/D (TM3), L188S (TM3), L194S (TM3), M243S (TM4), and 3xA (V262A-S263A-H264A), 3xG (V262G-S263G-H264G) (hinge region) (Figs. 3, *A* and *B*, S4*K*, S5*A*, and S5, *F–H*) besides the already known LoF mutations L81A, K85E (TM1) (54, 66), E149A/R (TM2) (60, 67), L174D (54) and V191N (57) (TM3) and L261D (54) (hinge region). In the following, we investigated the impact of these single point mutations first, on the coupling to STIM1-OASF and second, on their potential dominance over the robust MTR-GoF single point mutation, H134A. Among the LoF mutations discovered in Figure 3*A* only those located in the cytosolic, extended TM regions (CETR) (scheme, green ring) interfere with both STIM1 coupling (Fig. 3*C*) and the constitutive activity induced by the MTR-GoF H134A (Fig. 3*D*). Their plasma membrane expression remained unaffected as shown for Orai1 I148S, Orai1 E149K, and Orai1 S179F in Figure S5*E*. Conversely, other LoF single point mutations (*i.e.*, L188S, L194S) elucidated in Figure 3 a leave the coupling to STIM1 OASF and the constitutive activity of Orai1 H134A unimpaired (Fig. S5, *B*–*D*). Thus, loss of function of Orai1 L188S and Orai1 L194S cannot be explained by their approximately 30% reduced plasma membrane expression (Fig. S5*E*). The additional hinge mutations described above abolish the colocalization with STIM1 (Fig. S4*L*) but leave the constitutive activity of Orai1 H134A unaffected (Fig. S4, *K* and *M*).

Overall, CETR-LoF mutations possess a multidimensional role including inhibition of STIM1 binding as well as interference with an opening-permissive channel formation and function. Thus, we used these CETR-LoF mutations in the following to investigate their impact on different MTR-GoF mutations.

### N-terminal LoF point mutation K85E abolishes the constitutive activity of all GoF TM2/3/4 mutants

Initially, we investigated the impact of a defective N terminus on MTR-GoF point mutations employing the prominent N-terminal K85E point mutation leading to loss of function of Orai1 (66). We introduced K85E in a constitutive TM2-, TM3-, or TM4 point mutant in order to examine the impact of K85E and the respective GoF mutation on each other. All double/triple mutants: Orai1 K85E L130S, Orai1 K85E H134A, Orai1 K85E F136S (in TM2), Orai1 K85E L185A F250A, Orai1 K85E V181K, and Orai1 K85E V181A (in TM3/TM4) and Orai1 K85E P245L, Orai1 K85E S239C (in TM4) (Fig. S5, *I* and *J*) displayed loss of function also in the presence of STIM1, in line with Yeung *et al*. (57). Plasma membrane expression remained unaffected for all these inactive double/triple mutants (Fig. S5*L*). Coupling to the STIM1 C-terminal fragment OASF was partially reduced (Fig. S5, *M*–*P*), which, however, does not account for a total loss of function of the double/triple point mutants. In total, K85E is dominant over the GoF single point mutations and affects functional pore conformation and signal propagation already prior to STIM1 binding.

### CETR-LoF point mutations act dominant over MTR-GoF mutations independent of their location relative to each other

**Figure 4:**
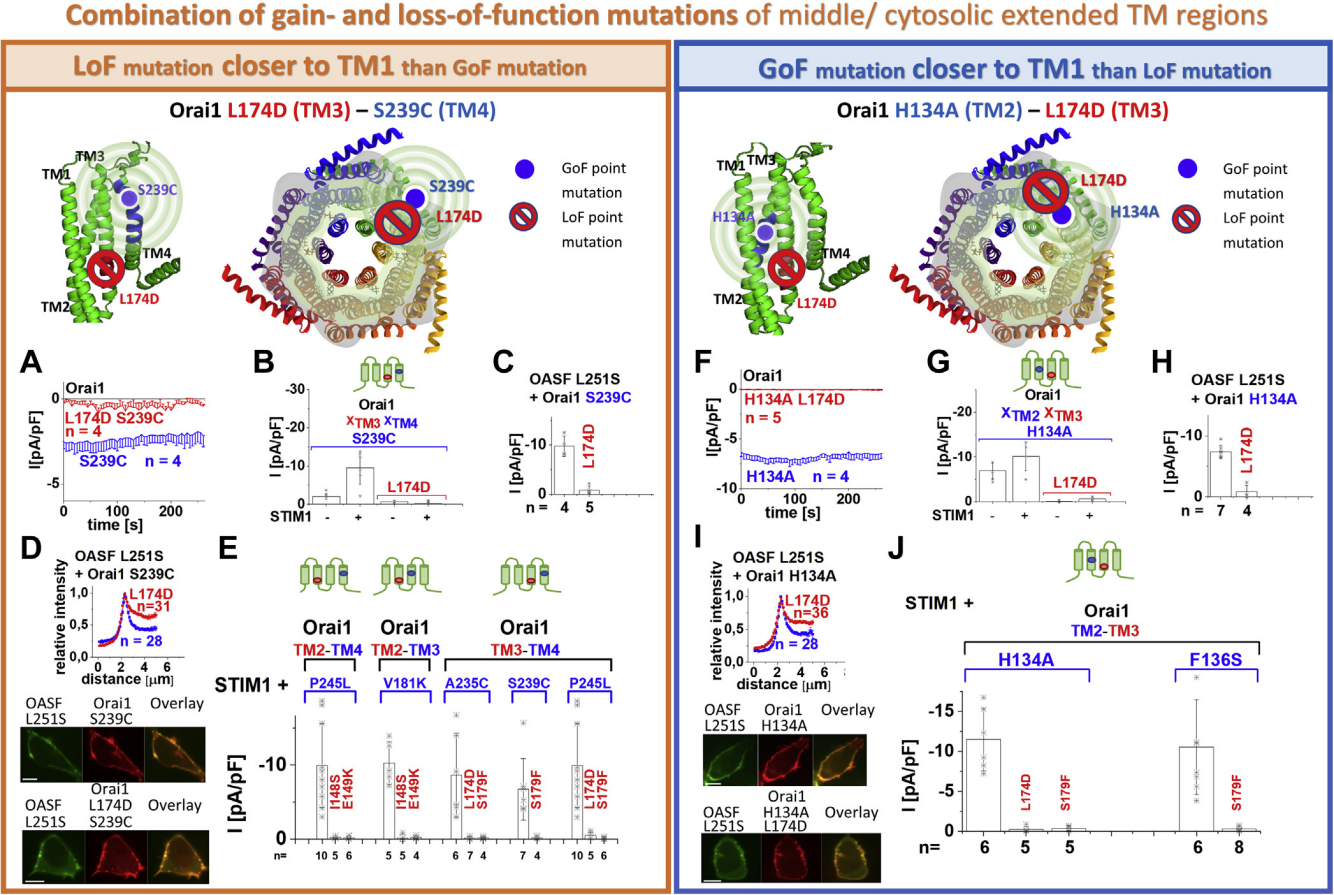
**Constitutive currents of MTR-GoF mutants are inhibited by CETR-LoF mutations in a dominant manner independent of their location relative to each other.** Four schemes representing the location of the investigated residues within a single subunit (*top left*) of Orai1 or the whole channel complex (*top middle*), for either LoF or GoF mutation closer to the pore, respectively. The *red stop sign* represents the position of the LoF mutation, while the *blue circle* shows the position of the GoF mutation. The *spheres* indicate the impact of the GoF mutation on the entire subunit. Special focus was addressed to H134, L174, and S239, owing to their location in the MTR close to pore (H134) or at the channel periphery (S239) or in the CETR (L174), respectively (*A–D*, *F–I*). By combining a GoF in the MTR and an LoF mutation in the CETR at the respective positions and investigating their impact on each other, we examined whether interdependent TM domain motions within the entire channel complex are necessary for pore opening. To provide a solid foundation for the conclusions obtained with the mutations at H134, L174, and S239, we investigated a diversity of other double mutants combining distinct LoF and GoF mutations at other positions (*E*, *J*). *A*, time courses of current densities after whole-cell break-in of Orai1 S239C compared with Orai1 L174D S239C in the absence of STIM1. Constitutive currents of the MTR-GoF Orai1 S239C mutant are inhibited by the additional introduction of the LoF mutation L174D. *B*, block diagram of whole-cell current densities of Orai1 S239C, Orai1 L174D S239C in the absence (t = 0 s) and the presence (maximum current densities) of STIM1 (n = 4–12 cells; values are mean ± SD). The current densities differed statistically significantly for the different Orai1 variants (Welch-ANOVA F(3, 7.05) = 13.25, *p* < 0.005. Games–Howell post hoc test revealed a significant difference between the GoF mutants and the corresponding GoF–LoF double mutants (*p* < 0.05). This holds for paired comparisons both in the absence, both in the presence, or one in the absence and one in the presence of STIM1. *C*, block diagram of whole-cell current densities of Orai1 S239C compared with Orai1 L174D S239C in the presence of STIM1 OASF L251S. Activation of the LoF–GoF Orai1 L174D S239C double mutant *via* STIM1 OASF L251S is significantly reduced compared with that of the GoF Orai1 S239C mutant (Mann–Whitney test *p* < 0.05). *D*, intensity plots of STIM1-OASF-L251S coexpressed with Orai1 S239C compared with Orai1 L174D S239C (at 4 μm, Mann–Whitney test *p* < 0.05). Image series depict YFP-Orai1 S239C (as quantitative analysis of STIM1-OASF-L251S localization is shown in the corresponding intensity plot in [*D*], the same image series like in Figure 2*D* is shown for Orai1 S239C) or YFP-Orai1 L174D S239C mutants, CFP-OASF L251S and overlay (the scale bar represents 10 μm). *E*, block diagram of maximum whole-cell current densities of Orai1 P245L compared with Orai1 I148S P245L and Orai1 E149K P245L (Welch-ANOVA F(2, 11.94) = 14.07, *p* < 0.001; Games–Howell post hoc test revealed significant difference for Orai1-GoF with each Orai1-GoF-LoF double point mutants [*p* < 0.05]); Orai1 V181K compared with Orai1 I148S V181K and Orai1 E149K V181K (Welch-ANOVA F(2, 6.27) = 27.95, *p* < 0.001; Games–Howell post hoc test revealed significant difference for Orai1-GoF with each Orai1-GoF-LoF double point mutants [*p* < 0.05]); Orai1 A235C in comparison with Orai1 L174D A235C and Orai1 S179F A235C (Welch-ANOVA F(2, 6.44) = 6.07, *p* < 0.05; Games–Howell post hoc test revealed significant difference for Orai1-GoF with each Orai1-GoF-LoF double point mutants [*p* < 0.05]); Orai1 S239C in comparison with Orai1 S179F S239C (Welch-ANOVA F(1, 6.12) = 17.34, *p* < 0.01; Games–Howell post hoc test revealed significant difference for Orai1-GoF with each Orai1-GoF-LoF double point mutants [*p* < 0.05]); Orai1 P245L in comparison with Orai1 L174D P245L and Orai1 S179F P245L (Welch-ANOVA F(2, 9.67) = 16.06, *p* < 0.001; Games–Howell post hoc test revealed significant difference for Orai1-GoF with each Orai1-GoF-LoF double point mutants [*p* < 0.05]), all in the presence of STIM1. *F*, time courses of current densities after whole-cell break-in of Orai1 H134A compared with Orai1 H134A L174D in the absence of STIM1. Constitutive currents of the MTR-GoF Orai1 H134A mutant are inhibited by the additional introduction of the LoF mutation L174D. *G*, block diagram of whole-cell current densities of Orai1 H134A, Orai1 H134A L174D in the absence and presence of STIM1 (n = 4–12 cells; values are mean ± SD). The current densities differed statistically significantly for the different Orai1 variants (Welch-ANOVA F(3, 6.70) = 32.48, *p* < 0.005; Games–Howell post hoc test revealed a significant difference between the GoF mutants and the corresponding GoF-LoF double mutants [*p* < 0.05]). This holds for paired comparisons both in the absence, both in the presence, or one in the absence and one in the presence of STIM1). *H*, block diagram of whole-cell current densities of Orai1 H134A compared with Orai1 H134A L174D in the presence of STIM1 OASF L251S. Activation of the LoF–GoF Orai1 H134A L174D double mutant *via* STIM1 OASF L251S is significantly reduced compared with that of the GoF Orai1 H134A mutant (Mann–Whitney test *p* < 0.05). *I*, intensity plots of STIM1-OASF-L251S coexpressed with Orai1 H134A compared with Orai1 H134A L174D (at 4 μm, Mann–Whitney *p* < 0.05). Image series depict YFP-Orai1 H134A (as quantitative analysis of STIM1-OASF-L251S localization is shown in the corresponding intensity plot in (*I*), the same image series like in Figure 2*I* is shown for Orai1 H134A) or YFP-Orai1 H134A L174D mutants, CFP-OASF L251S, and overlay (the scale bar represents 10 μm). *J*, block diagram of maximum Orai1 mutant whole-cell current densities of Orai1 H134A compared with Orai1 H134A L174D, Orai1 H134A S179F (Welch-ANOVA F(2, 11.79) = 8.37, *p* < 0.01; Games–Howell post hoc test revealed significant difference for Orai1-GoF with each Orai1-GoF-LoF double point mutants [*p* < 0.05]) and Orai1 F136S compared with Orai1 F136S S179F (Welch-ANOVA F(1, 9.04) = 13.71, *p* < 0.01; Games–Howell post hoc test revealed significant difference for Orai1-GoF with each Orai1-GoF-LoF double point mutants [*p* < 0.05]), all coexpressed with STIM1. CETR, cytosolic extended transmembrane region; GoF, gain of function; LoF, loss of function; MTR, middle transmembrane region.

Next, we employed Orai1 CETR-LoF mutations of TM2 and TM3 to investigate their impact on the above described Orai1 GoF mutations located in the MTR. Therefore, we combined LoF and GoF point mutations in distinct TM domains, whereas one was located closer to the pore (*e.g.*, TM2) than the other one (*e.g.*, TM3), with special focus on H134, S239 in the MTR and L174 in the CETR. The reason for the special focus on H134 or S239 in combination with L174 is to investigate the influence of a LoF and a GoF mutation on each other, which extends from the MTR to the CETR. As expected, the double mutant Orai1 L174D S239C, containing the CETR-LoF-L174D in TM3 closer to the pore than the MTR-GoF-S239C in TM4, exhibited loss of function, both in the absence and presence of STIM1 (Fig. 4, *A* and *B*). Analogously, other constitutive mutants with a CETR-LoF point mutation located closer to TM1 than the MTR-GoF substitution (Orai1 I148S V181K, Orai1 E149K V181K, Orai1 I148S/E149K P245L, Orai1 L174D/S179F P245L, Orai1 L174D A235C; Fig. 4*E*) displayed also loss of function. Of interest, vice versa also double mutants with the MTR-GoF point mutation closer to TM1 (*e.g.*, H134A in TM2) than the CETR-LoF point mutation (*e.g.*, L174D in TM3), such as Orai1 H134A L174D, and others (Orai1 H134A S179F, Orai1 F136S S179F) displayed an abolished constitutive activity independent of the presence of STIM1 (Fig. 4, *F*, *G*, and *J*). Moreover, we combined CETR-LoF and MTR-GoF point mutations of the two membrane planes within one TM domain (Orai1 H134A I148S/E149K in TM2, Orai1 L174D L185A F250A in TM3). These mutants also failed to show constitutive activity (Fig. S3, *R* and *T*). All nonfunctional double mutants displayed comparable plasma membrane localization like the constitutive single point mutant (Fig. S3, *B*, *J*, *O*, and *W*). Also, the constitutive activity of the Orai1 ANSGA mutant can be overruled by LoF point mutations in the CETR, similar to the loss of function of Orai1 K85E ANSGA (54). Collectively, similar to the MTR-LoF in Figure 2, also CETR-LoF mutations act dominant over MTR-GoF mutations.

In line with the findings above, we observed upon coexpression of STIM1 OASF L251S a total loss of function of both Orai1 L174D S239C and Orai1 H134A L174D compared with the constitutively active, single point mutants Orai1 S239C and Orai1 H134A, respectively (Fig. 4, *C* and *H*). Investigation of the coupling of STIM1 *via* OASF L251S and OASF revealed significantly reduced, but not completely abolished, coupling of STIM1 to Orai1 L174D S239C and Orai1 H134A L174D (Figs. 4, *D* and *I* and S3, *I* and *N*). Also, other double point mutants exhibit significantly reduced coupling to those STIM1 fragments as their corresponding constitutively active point mutants (Fig. S3, *G*–*I*, *M*, *N*, *S*, and *U*).

Also in this case, V102A in TM1 represents an exception. We discovered that none of the above reported CETR-LoF mutations as exemplarily tested with I148S, E149K and S179F were able to abolish the constitutive function of Orai1 V102A. These findings are also in line with the published activity of Orai1 V102A L174D (54). Coexpression of STIM1 was not able to enhance V_rev_ of V102A double mutants containing the cytosolic LoF point mutants (Fig. S4, *C*–*H*), indicating that these CETR mutations hinder STIM1 coupling. In contrast, the constitutive activity of two other GoF-TM1 mutants, F99M and V107M, could be abolished upon the introduction of the CETR-LoF E149K mutation (Fig. S4, *I* and *J*).

Summarizing, a series of MTR-GoF-CETR-LoF double point mutants showed that also LoF point mutations in the CETR act dominant over GoF point mutations, except for the GoF-V102A. Thus, mutually dependent motions of the TM regions occur not only in one membrane plane within the MTR but also across several membrane planes as here the MTR and CETR. Herewith, we provide fundamental evidence that a conformational alteration of a single checkpoint residue spreads across the entire Orai1 subunit. Moreover, the global conformational change is indispensable for pore opening.

### LoF point mutations keep the pore of a constitutively active Orai1 mutant in a closed conformation

**Figure 5:**
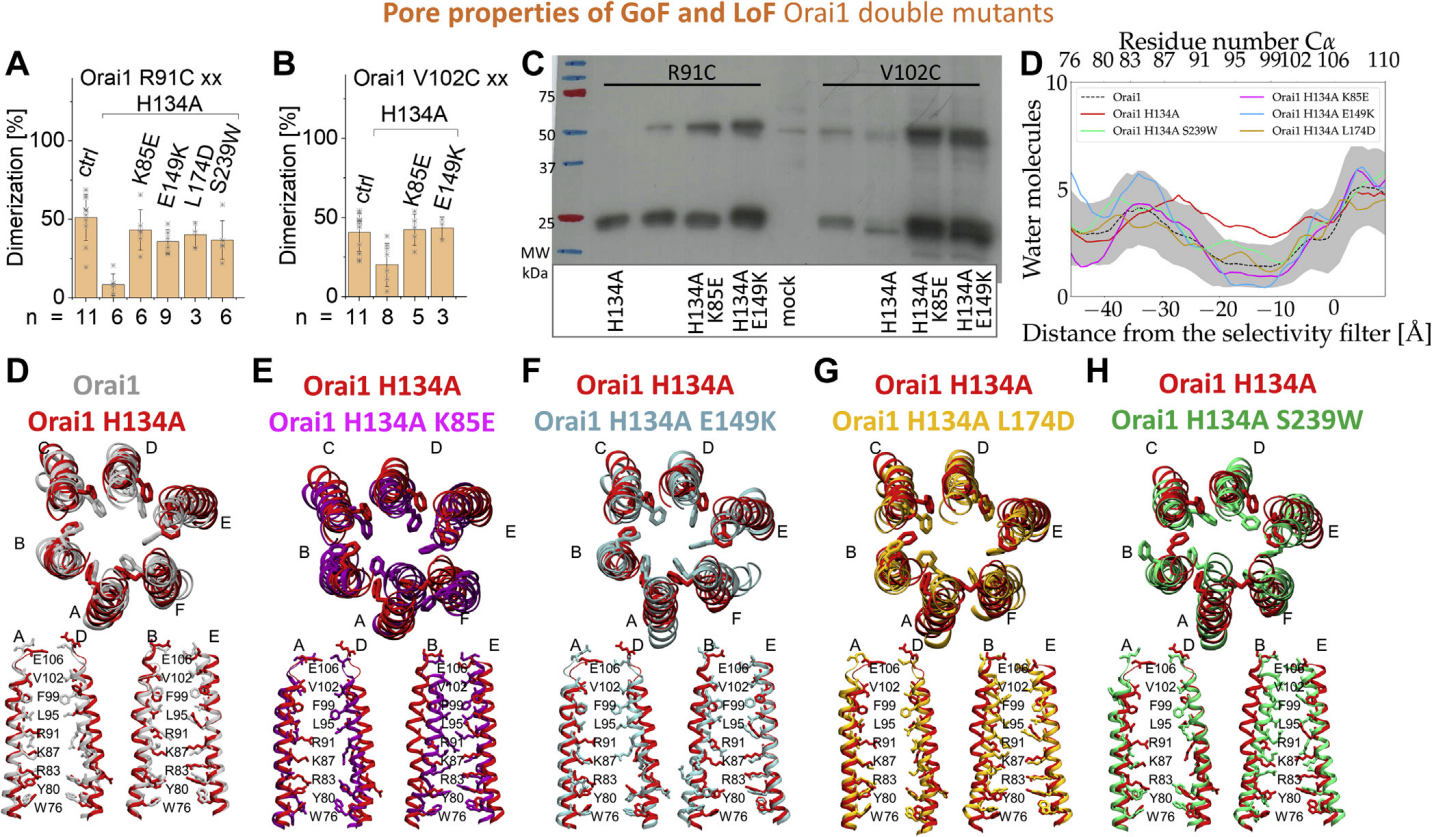
**Orai1 middle transmembrane region– and cytosolic extended transmembrane region–LoF point mutations hinder the formation of an opening-permissive pore geometry.***A*, *B*, in order to determine alteration in the pore conformation, we performed cysteine cross-linking of the pore lining residues R91 and V102 for the GoF-Orai1 H134A mutant compared with different LoF–GoF Orai1 double mutants. Block diagrams exhibiting the degree of dimerization obtained *via* cysteine cross-linking of Orai1 R91C in comparison with Orai1 R91C H134A, Orai1 K85E R91C H134A, Orai1 R91C H134A E149K, Orai1 R91C H134A L174D, and Orai1 R91C H134A S239W (*A*) and Orai1 V102C in comparison with Orai1 V102C H134A, Orai1 K85E V102C H134A, and Orai1 V102A H134A E149K (*B*). Extent of cross-linking differed statistically significantly for the different mutants (Welch-ANOVA F(5, 10.64) = 16.08, *p* < 0.001 [*A*], F(3, 8.97) = 4.97, *p* < 0.05 [*B*]). Games–Howell post hoc test revealed significant difference for the single and triple mutants compared with the double mutant (*p* < 0.05). *C*, representative Western blots for (*A*) and (*B*), respectively. Apparently, a reduced amount of surviving cells, owing to expression of constitutively active mutants, is reflected by lighter bands. *D*, pore hydration profile for wildtype Orai1 and mutants. The number of water molecules is given as a function of the distance from the selectivity filter. Hydration profiles are given for wildtype in *dotted black line* where the *gray shaded areas* correspond to the standard deviation of the mean. *Solid red*, *purple*, *blue*, *orange*, and *green lines* correspond to Orai1 H134A, Orai1 K85E H134A, Orai1 H134A E149K, Orai1 H134A L174D, and Orai1 H134A S239W. The profiles were calculated using the last 50 ns of the simulations. Positions of the carbon α of the residues delineating the pore are given on the top axis, while the distance from the selectivity filter is given on the bottom axis. *E*, molecular dynamics simulations demonstrate increased pore helix rotation and pore hydration in GoF mutation H134A. Superposition of snapshots at *t* = 400 ns from molecular dynamics simulations of WT (*gray*) and H134A (*red*) as viewed from the top. Pairs of diagonal subunits viewed from the side (*bottom*). *E–H*, superposition of snapshots at t = 400 ns from molecular dynamics simulations of H134A (*red*) and K85E H134A (*purple*) (*E*), Orai1 H134A E149K (*blue*) (*F*), Orai1 H134A L174D (*orange*) (*G*), Orai1 H134A S239W (*green*) (*H*) as viewed from the top. Pairs of diagonal subunits viewed from the side (*bottom*). GoF, gain of function; LoF, loss of function.

The above-described dominant effect of diverse LoF over GoF mutations indicate that the LoF mutations keep the constitutively active Orai1 complex in the closed conformation. To verify that the Orai1 pore architecture of constitutively active mutants is affected by those LoF point mutations, we performed cysteine cross-linking and molecular dynamics (MD) simulations on the following key double mutants Orai1 K85E H134A, Orai1 H134A E149K, Orai1 H134A L174D, and Orai1 H134A S239W in comparison with Orai1 H134A. Frischauf *et al*. (51) have recently demonstrated that cysteine cross-linking along the critical pore-lining residues, Orai1 R91C and Orai1 V102C, is significantly reduced upon the introduction of H134A. In contrast, the cross-linking of those positions upon introduction of the double mutations K85E H134A, H134A E149K, H134A L174D (only for Orai1 R91C) and H134A S239W (only for Orai1 R91C) exhibited again significantly enhanced levels of cysteine cross-linking compared with the absence of the H134A mutation (Fig. 5, *A* and *B*).

In line with the findings by Yeung *et al*. (57), our MD simulations up to 400 ns long revealed for Orai1 H134A in comparison with Orai1 wildtype enhanced hydration of the pore together with a rotation of F99 out of the pore helix and a dilation of the pore. According to our functional data, the hydration of the pore of the double mutants Orai1 K85E H134A, Orai1 H134A E149K, Orai1 H134A L174D and Orai1 H134A S239W (Fig. 5*C*) was significantly reduced compared with Orai1 H134A. Although Orai1 H134A L174D and Orai1 H134A S239W reached hydration levels comparable with that of the Orai1 wildtype, Orai1 K85E H134A and Orai1 H134A E149K showed significantly enhanced dehydration also compared with the Orai1 wildtype. Moreover, an investigation of the associated conformational changes revealed that F99 turned back to point again more into the pore region similar to the wildtype Orai1 (Fig. 5, *D*–*H*).

In summary, these data reveal that both CETR- as well as MTR-LoF mutations independent of their location relative to the GoF in the TM domains either closer or farther apart and in distinct membrane planes (MTR, CETR) hamper pore dilation induced *via* a GoF mutation.

### Local enrichment of STIM1 overrules the dominant effect of MTR- but not of CETR-LoF mutations

**Figure 6:**
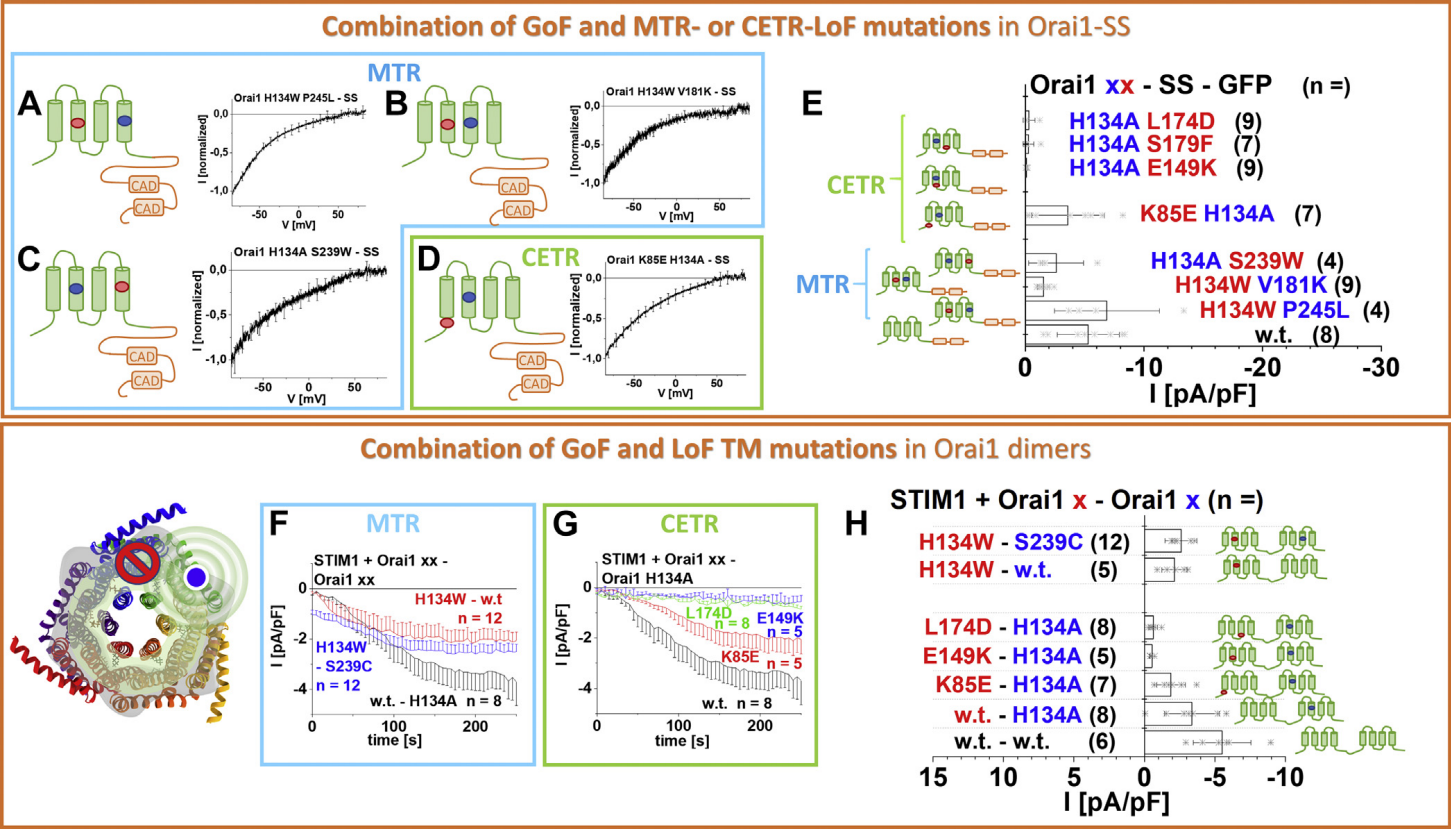
**CETR-LoF mutations are dominant over MTR-GoF mutations, both upon local enrichment of STIM1-CAD and within an Orai1 dimer.***A–D*, current–voltage relationships of Orai1 H134W P245L–SS–GFP, Orai1 H134W V181K–SS–GFP, Orai1 H134A S239W–SS–GFP, and Orai1 K85E H134A–SS–GFP representing the effect of MTR-LoF mutations (*blue*; *A–C*) or the effect of CETR-LoF mutation (*green*; *D*). *E*, block diagram of maximum whole-cell current densities of Orai1–SS–GFP wildtype in comparison with Orai1 H134A L174D–SS–GFP, Orai1 H134A S179F–SS–GFP, Orai1 H134A E149K–SS–GFP, Orai1 K85E H134A–SS–GFP, Orai1 H134A S239W–SS–GFP, Orai1 H134W V181K–SS–GFP, and Orai1 H134W P245L–SS–GFP. Current densities of mutants containing E149K, S179F, or L174D are significantly different compared with the wildtype construct. (Welch-ANOVA F(7, 15.49) = 15.19, *p* < 0.001. Games–Howell post hoc test revealed a significant difference between Orai1–SS–GFP and Orai1 H134A L174D–SS–GFP, Orai1 H134A S179F–SS–GFP, as well as Orai1 H134A E149K–SS–GFP, respectively [*p* < 0.05]). *Green encircled schemes* represent the CETR LoF mutations, while *blue encircled schemes* show MTR-LoF mutations. Schemes display the location of one of the representative combinations of investigated residues within the whole Orai1 channel complex (*bottom left*). The *red stop sign* represents the position of the LoF mutant, while the *blue circle* shows the position of GoF mutant. The *spheres* indicate the impact of the GoF mutation on the entire subunit and an adjacent subunit. *F*, *G*, time course of Orai1 dimer current densities after whole-cell break-in in the presence of STIM1. Orai1 dimer mutants represent Orai1–Orai1 H134A compared with Orai1 H134W–Orai1 and Orai1 H134W–Orai1 S239C showing the effect of an MTR-LoF mutation (*blue*) (*F*). Orai1 dimer mutants represent Orai1–Orai1 H134A in comparison with Orai1 K85E–Orai1 H134A, Orai1 E149K–Orai1 H134A, and Orai1 L174D–Orai1 H134A showing the effect of a CETR LoF mutation (*green*) (*G*). *H*, block diagram of maximum whole-cell current densities of Orai1 dimers in the presence of STIM1 corresponding to current densities recorded in (*F*) and (*G*) compared with the dimer formed by WT Orai1 (Orai1–Orai1). Current densities of mutants containing E149K, S179F, or L174D differed statistically significantly compared with those of the wildtype construct. The current densities for the different Orai1-dimer variants (Welch-ANOVA F(6, 17.19) = 15.34, *p* < 0.001; Games–Howell post hoc test revealed a significant difference between STIM1-mediated currents of Orai1–Orai1 and Orai1 K85E–Orai1 H134A as well as Orai1–Orai1 or Orai1–Orai1 H134A and Orai1 E149K–Orai1 H134A as well as Orai1 L174D–Orai1 H134A, respectively [*p* < 0.05]). *A–E* and *H*, the schemes indicate the position of the corresponding mutations. The *blue circle* indicates GoF mutation, while the *red circle* indicates the LoF mutation. CETR, cytosolic extended transmembrane region; GoF, gain of function; LoF, loss of function; MTR, middle transmembrane region.

In the following, we tested whether local enrichment of STIM1 C-terminal fragments, induced by attaching two CAD fragments (-SS) to the Orai1 C terminus of the double point mutants, is able to overcome the dominant role of LoF point mutations. Indeed, these close-by STIM1 fragments were able to overrule the dominant role of MTR-LoF over MTR-GoF point mutations, as visible by the constitutive activity of Orai1 H134W P245L–SS, Orai1 H134W V181K–SS and Orai1 H134A S239W–SS (Fig. 6, *A*–*C* and *E*).

Also, the local attachment of two CAD fragments at the C terminus of Orai1 K85E H134A (Orai1 K85E H134A–SS) is able to counteract the inhibitory effect of K85E even better as for Orai1-K85E-SS (Fig. S5*Q*), which shows only small constitutive activity (Fig. 6, *D* and *E*). In contrast, double point mutants containing the MTR-GoF-H134A and another CETR-LoF mutation remain inactive upon local attachment of STIM1 fragments at their C termini (Orai1 H134A E149K–SS, Orai1 H134A S179F–SS, Orai1 H134A L174D–SS; Fig. 6*E*).

Overall, we discovered for MTR-GoF-LoF double mutants and Orai1 K85E-H134A that the pore opening can still be re-established by enhancing local concentrations of STIM1. In contrast, other CETR-LoF point mutations seem to severely affect both the pore geometry and STIM1 binding.

### CETR-LoF mutants are dominant over GoF mutants in Orai dimers

So far, we investigated the effects of LoF mutations in single Orai1 subunits. In the following, we were further interested whether the above-described critical LoF mutations affect also GoF mutations of the neighboring subunits.

Initially, we generated dimers with one wildtype subunit and one subunit containing the constitutive MTR-GoF-H134A (Fig. 6*H*) or -P245L (data not shown) mutation. In contrast to the strong constitutive activity of these point mutations in the homomer, in the heteromer, they allow only store-operated activation in the presence of STIM1 (Fig. 6*H*) in line with the store-dependent activation of an Orai1 dimer containing one wildtype and one Orai1 V102A subunit (68). This indicates that at least more than three GoF mutations within a hexamer are required to induce constitutive activity.

In the following, we generated a variety of Orai1 dimers containing an LoF mutation, in the first subunit and a GoF mutation in the second subunit. All of them remained inactive in the absence of STIM1, whereas in the presence of STIM1 only some exhibited activation. In line with preserved STIM1-mediated activation of Orai1 H134W-Orai1, also Orai1 H134W-Orai1 S239C exhibited store-dependent activation upon coexpression with STIM1 to a similar extent as for Orai1-Orai1 H134A (Fig. 6, *F* and *H*). Of interest, also Orai1 K85E-Orai1 H134A displayed store-operated currents (Fig. 6*G*), in accordance with the activity of the Orai1 K85E-Orai1 dimer published in Cai *et al*. (68). In contrast, Orai1 E149K–Orai1 H134 A and Orai1 L174D–Orai1 H134A remained inactive in the presence of STIM1 (Fig. 6, *G* and *H*).

To sum up, these data show that at least in the presence of STIM1 only CETR-LoF mutations except K85E act dominant over a GoF mutation in the neighboring subunit of a dimer and, thus, overall in the whole channel complex in an allosteric manner. We demonstrate that interdependent motions initiated by an activation signal to induce pore opening spread not only in individual subunits but also across neighboring subunits. The positions E149 and L174 are indispensable for this interdependent communication.

### Inter- and intra-Orai1 salt-bridge interactions in the CETR establish an intact STIM1-mediated Orai1 activation

Owing to the more dominant role of the CETR-LoF mutations and to gain some insights into how K85E and E149K mutations can induce loss of function, we continued to investigate their role *via* MD simulations for selected mutants and extensive site-directed mutagenesis.

**Figure 7:**
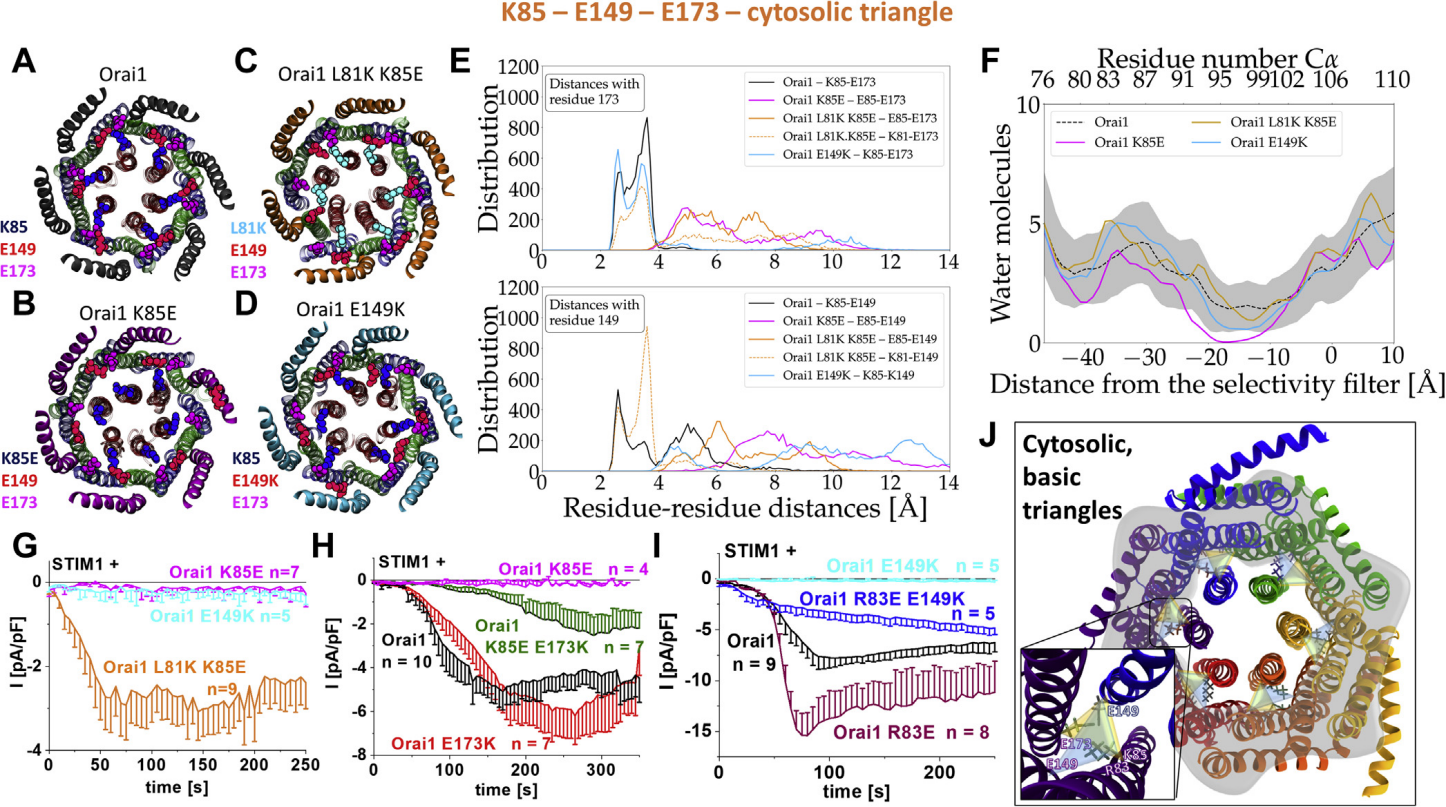
**The cytosolic triangles contribute to the establishment of an opening-permissive pore geometry and controls maintenance of STIM1-mediated Orai1 activation.***A*–*D*, cytosolic view of the starting and final snapshots of wildtype Orai1 (*A*), Orai1 K85E (*B*), Orai1 L81K K85E (*C*), and Orai1 E149K (*D*) from molecular dynamics simulations. TM1, TM2, and TM3 are represented in glassy ribbon material in *red*, *cyan*, and *green* respectively. TM4 is represented in a *yellow opaque ribbon*. Residues L81K, K85(E85), E149(K149), and E173 are colored *cyan*, *blue*, *red*, and *violet*, respectively. Channel conformation is shown after 250 ns of molecular dynamics, respectively. Differently colored C termini mark the respective mutant with the color code also used in (*E*). *E*, distribution of the distances calculated between the nitrogen atom of the lysine and the oxygen atoms from the carboxyl group of the glutamic acids for the last 50 ns of the simulations shown for the wildtype Orai1 (*black*), Orai1 K85E (*purple*), Orai1 L81K K85E (*orange*, *dotted orange*), and Orai1 E149K (*cyan*) (distance for E173 [*top*], distance for E149 [*bottom*]). *F*, pore hydration profile for wildtype Orai1 and mutants. The number of water molecules is given as a function of the distance from the selectivity filter. Hydration profiles are given for wildtype in *dotted black line* where the *gray shaded areas* correspond to the standard deviation of the mean. *Dashed black*, *blue*, *purple*, and *orange lines* correspond to Orai1 wildtype, Orai1 E149K, Orai1 K85E, and Orai1 L81K K85E. The profiles were calculated using the last 50 ns of the simulations. Positions of the carbon α of the residues delineating the pore are given on the top axis with distance from the selectivity filter shown on the bottom. *G*–*I*, electrophysiological characterization of STIM1-mediated currents of Orai1 mutants containing 1 or 2 residues in the cytosolic triangle mutated. Time course of STIM1-mediated whole-cell current densities of Orai1 E149K, Orai1 K85E, and Orai1 L81K K85E. Only Orai1 L81K K85E, but not Orai1 E149K and Orai1 K85E, show store-operated current activation in the presence of STIM1 similar to CRAC channel activation (comparison of maximum current densities revealed with Welch-ANOVA F(2, 11.17) = 7.61, *p* < 0.01; Games–Howell post hoc test revealed a significant difference between Orai1 L81K K85E and Orai1 K85E as well as Orai1 E149K, respectively [*p* < 0.05]) (*G*). Time course of STIM1-mediated whole-cell current densities of Orai1 K85E, Orai1 E173K, and Orai1 K85E E173K in comparison with wildtype Orai1. STIM1-mediated activation of Orai1 E173K amounts to similar levels like that of wildtype Orai1. Although Orai1 K85E shows loss of function, the Orai1 K85E E173K double mutant exhibits restored activity (comparison of maximum current densities revealed with Welch-ANOVA F(3, 11.78) = 43.19, *p* < 0.001; Games–Howell post hoc test revealed a significant difference between Orai1 wildtype and Orai1 K85E as well as Orai1 K85E E173K, respectively [*p* < 0.05]) (*H*). Time course of STIM1-mediated whole-cell current densities of Orai1 R83E, Orai1 E149K, and Orai1 R83E E149K in comparison with wildtype Orai1. STIM1-mediated activation of Orai1 R83E reaches higher levels than that of wildtype Orai1. Although Orai1 E149K shows loss of function, the Orai1 R83E E149K double mutant exhibits restored activity (comparison of maximum current densities revealed with Welch-ANOVA F(3, 9.73) = 142.12, *p* < 0.001; Games–Howell post hoc test revealed a significant difference between Orai1 and Orai1 R83E as well as Orai1 E149K, respectively [*p* < 0.05]) (*I*). *J*, the scheme with inset represents the two cytosolic triangles formed by the residues K85, E149, E173 and R83, E149, E173, respectively. TM, transmembrane domain.

In MD simulations using the closed hOrai structure, we initially focused on the residues R83, K85, E149, and E173 and compared the axial positions of the carbon alpha relative to the hydrophobic gate as well as the distances between the charged moieties of each residue. The carbon alpha from K85 and E173 are on the same height, whereas the carbon alpha of R83 is located further toward the cytosol followed by the carbon alpha from E149 (Fig. S6*A*, top). Although the positions of the charged moieties from K85 and E173 follow the same observations, guanidine and carboxylate moieties from R83 and E149, respectively, are now at the same level as well (Fig. S6*A*, bottom). By evaluation of the distances between the four residues, we clearly observed that K85 and E173 within the same subunit and K85 and E149 of adjacent subunits have their side chains converging toward each other, thus, potentially forming salt-bridge interactions (Figs. 7*A* and S6*B*). Although K85–E173 are in close proximity with distances ranging between 2 and 4 Å, the pair K85–E149 comes in more than 50% of the cases closer than 4 Å (Fig. 7*E*). On the contrary, R83–E149 and R83–E173 are less likely to form salt bridges as their side chains are oriented in the same direction or away from each other, at least in the structure of resting, closed Orai1 (Fig. S6*C*). Despite this observation, we noticed that TM1 offers enough flexibility in the basic region to allow for a slight rotation, allowing R83 to interact with E149 within one subunit (Fig. S6*C*), in line with Dong *et al*. (60).

The K85E mutation led to the disappearance of these salt-bridge interactions in Orai1 (Fig. 7, *B* and *E*). K85E drifted slowly away from E149 and E173 (Fig. S6*B*), which finally led to a collapse of the pore (Fig. 7*F*). The hydration profiles (Figs. 7*F* and S6, *D*–*F*) corroborated these observations as the number of water molecules within the pore region of the channel significantly dropped in the case of Orai1 K85E. MD simulations on the Orai1 E149K revealed that especially the salt bridge of K85–E149 was lost (Fig. 7, *D* and *E*), while the mean distance of K85–E173 slightly enlarged (Fig. S6*B*). This change was sufficient to also lead to a reduced amount of water molecules in the pore, concomitantly, with a clockwise rotation of F99 (Fig. S6, *D*–*F*).

Introduction of a positively charged residue, L81K (Fig. 7, *C* and *G*), one helical turn N-terminal to K85E (Orai1 L81K K85E) restored or, at least, maintained the original pore structure since its hydration level is close to that of the wildtype (Fig. 7*F*). In line, the salt bridge bonds with E149 of the neighboring subunit and E173 within one subunit naturally occurring in the wildtype and destroyed by the K85E mutation are restored in Orai1 L81K K85E (Fig. 7, *C*, *E*, and *F*). Accordingly, a screen on several N-terminal mutations (L81K, S82K, A88K, and S89K) revealed that STIM1-mediated activation and coupling of Orai1 K85E was reconstituted only by the additional introduction of L81K (Orai1 L81K K85E) (Figs. 7*G* and S4*A*).

For further clarification of the impact of potential salt-bridge interactions of K85–E173 and K85–E149 in Orai1 activation, we tested double mutants with the respective residues exchanged by ones with oppositely charged moieties. We uncovered that Orai1 K85E E173K recovers STIM1-mediated activation partially (Fig. 7*H*), in line with Dong *et al*. (60). In contrast, we find that Orai1 K85E E149K remains nonfunctional (Fig. S7*A*), although our simulations clearly indicate a close distance and electrostatic interaction of K85–E149 (Fig. 7, *A* and *E*). The reason for that is probably that only in Orai1 K85E E149K, but not in Orai1 K85E E173K, the charged moieties at the respective positions move farther apart compared with the wildtype. It is worth noting that the indispensable role of the K85–E149 pair clearly stands out when comparing single point mutants at the positions R83, K85, E149, and E173 substituted to a residue with an oppositely charged side chain. Although Orai1 R83E remains functional, Orai1 E149K loses function, although in both mutants the R83–E149 interplay is lost (Fig. S7*A*). Moreover, Orai1 E173K shows normal activation, whereas K85E remains inactive, although in both mutants the K85–E173 salt bridge is lost (Fig. S7*A*). The difference can be explained by the K85–E149 pair, which remains intact in the functional mutants Orai1 R83E and Orai1 E173K, but not in the LoF mutants Orai1 K85E and Orai1 E149K.

In search of further electrostatic interaction partners of E149, we identified R83 as a crucial residue. Orai1 R83E E149K displays restored STIM1-mediated activation (Fig. 7*I*), in line with Dong *et al*. (60), who suggested that R83 can rotate clockwise toward E149. We termed these crucial salt-bridge interactions established within and between adjacent subunits the cytosolic triangles (Fig. 7*J*). This salt-bridge pair seems to be especially crucial during activation *via* STIM1. In support of the role of STIM1, we showed *via* the GoF Orai1 H134A mutant that the triple mutant Orai1 R83E H134A E149K remains inactive in the absence of STIM1 but becomes active in the presence of STIM1 (Fig. S7, *B* and *C*).

**Figure 8:**
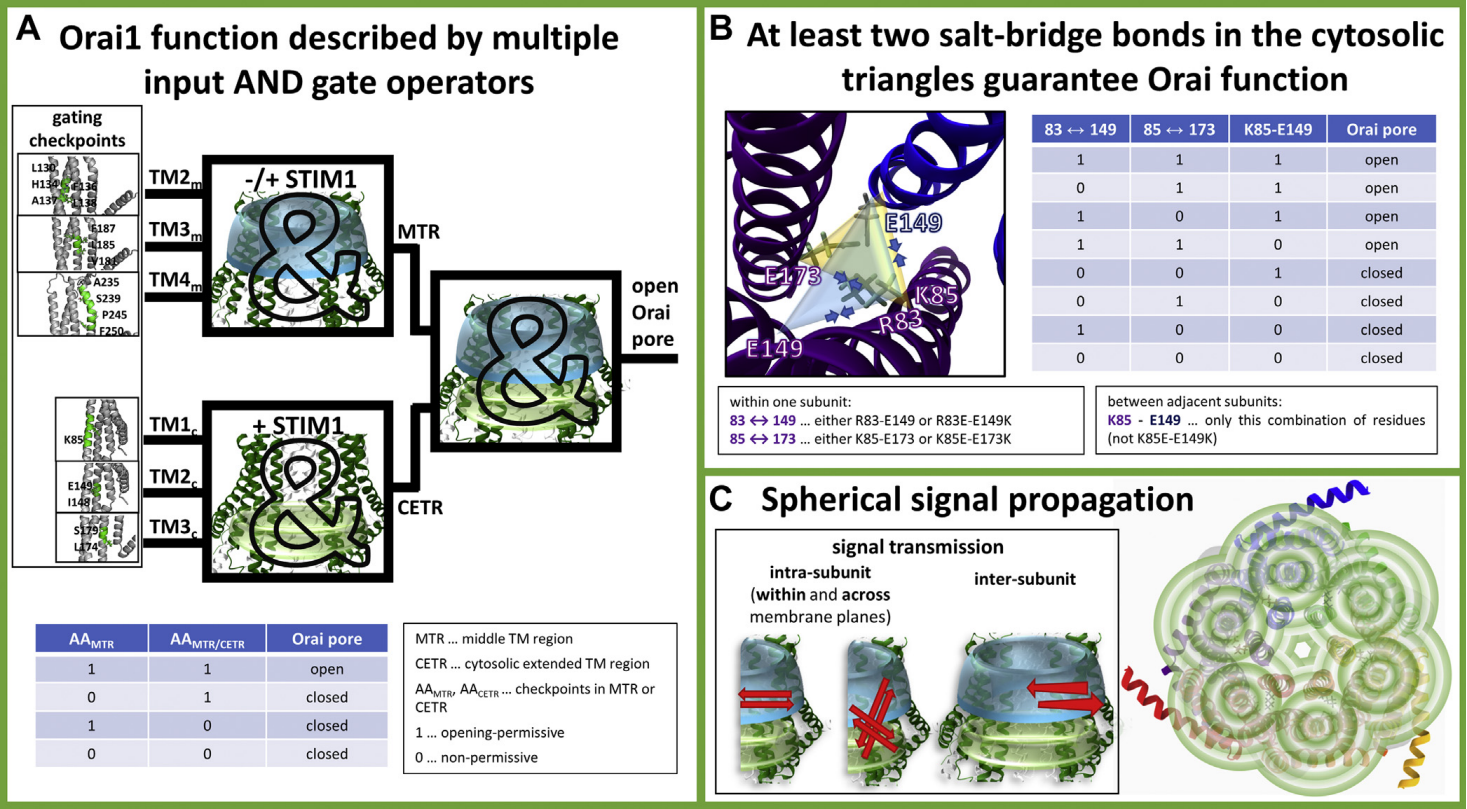
**Orai1 signal propagation.***A*, Orai pore opening requires a series of gating checkpoints to capture an opening-permissive conformation. A single residue in a nonpermissive conformation impairs pore opening. These complex correlations can be best described by the operator symbol for the AND gate containing multiple inputs, used in digital electronics. These multiple inputs include gating checkpoints in the MTR and the CETR. Each section individually, and also together, can be viewed as an AND gate. The truth table (*bottom*), simplified for just two gating checkpoints AA_MTR_ and a second AA_MTR_ or an AA_CETR_ shows that as soon as one of two gating hots adopts a nonpermissive conformation, the Orai channels remain closed. *B*, the CETR includes the cytosolic triangles that form salt-bridge interactions within and between subunit(s). Three pairs of charged residues R83–E149, K85–E173, and K85–E149 are functionally relevant. Among those at least two salt bridges are required to be intact and maintain STIM1-mediated Orai1 activation. These requirements can be described by the truth table in (*B*) and thus their interplay can be illustrated by an OR gate of always two salt bridges (Fig. S9*B*) that maintain an opening-permissive channel conformation. To maintain Orai1 function only the pairs R83–E149 and K85–E149, but not the K85–E149, can be mutated to oppositely charged residues. *C*, an Orai1 activation signal either from a constitutively active mutation or from STIM1 coupling to Orai1 C terminus impacts each subunit (*via* alteration of intrasubunit interactions) within and across membrane planes (MTR, CETR) and thus, the entire channel complex (*via* alteration in intersubunit interaction). CETR, cytosolic extended transmembrane region; MTR, middle transmembrane region.

We extended our functional screen to additional multiple point mutants with diverse combinations of these basic and acidic residues substituted (Fig. S7*A*). Accordingly, we evaluated the number of potential attracting and repulsing forces of the residues at positions 83, 85, 149, and 173 (Fig. S7*A*) in functional compared with nonfunctional mutants. This combined approach unveils that two intrasubunit salt bridges, R83–E149, K85–E173, and one intersubunit salt bridge, K85–E149, play an essential role in maintaining Orai1 activation. Moreover, at least two of these communication pairs need to be intact to maintain Orai1 activation (Figs. 8*B*, S7*A*, and S9).

In accordance with the functional data, we discovered that the salt-bridge interactions in the CETR also impact STIM1 coupling. Although the plasma membrane intensity of the STIM1 C-terminal fragments, OASF and OASF L251S, was strongly reduced when coexpressed with these single point mutants K85E and E149K, the double point mutants Orai1 K85E E173K and Orai1 R83E E149K displayed again restored coupling of STIM1 (Fig. S8, *A*–*D*).

In conclusion, the sophisticated interplay of the basic and acidic residues in the CETR is a core element for CRAC channel function, pore hydration, pore opening, and STIM1 coupling. Our results indicate that STIM1-induced Orai1 activation promotes salt-bridge interactions in the CETR, especially that of R83–E149 within one subunit, to ensure an intact pore geometry. We termed these crucial salt-bridge interactions established within and between adjacent subunits the cytosolic triangles (Fig. 7*J*).

### Orai1 CETR-LoF L174D at the hinge plate does not interfere with the cytosolic triangle

Orai1 L174D, a recently reported LoF mutant in the hinge plate (54), has been proposed to affect the communication with L261 in TM4 and in consequence signal propagation to the pore. As shown in Figure S10*A*, MD simulations revealed that L174D affects the hydrophobic gate of Orai1 in a similar way to K85E and E149K.

In a further step, we investigated whether in Orai1 L174D, the negatively charged aspartate interferes with the salt-bridge interactions between TM1-TM2-TM3, owing to its close positioning to E173. However, MD simulations revealed that Orai1 L174D widely maintains the K85–E173 and K85–E149 salt bridge interactions in both Orai1 and Orai1 H134A (Fig. S10*B*). Our following data indicate in line with Zhou *et al*. (54) that L174 in TM3 maintains Orai1 function *via* communication with L261 in TM4. Although we were unable to restore STIM1-mediated Orai1 activation *via* the introduction of oppositely charged residues at L174 and L261 (Orai1 L174D L261K, Orai1 L174K L261D; Fig. S10*C* right), we discovered that a double point mutant Orai1 E173K L261D displays pronounced activity in contrast to the strongly reduced currents of Orai1 L261D (Fig. S10*C*). A triple mutant Orai1 K85E E173K L261D displays again a loss of store-operated activation in the presence of STIM1 (Fig. S10*C*, right), despite maintaining the function of Orai1 K85E E173K (Fig. 7*H*).

In summary, besides the triangular interaction of basic and acidic residues in the CETR, the interplay of hydrophobic leucines in TM3 and TM4 represent a second individual module required for an intact Orai1 channel. The L174D mutation allosterically affects the pore in a similar manner like Orai1 K85E and Orai1 E149K but does not interfere with the salt-bridge interactions of the cytosolic triangles.

## Discussion

In this study we provide profound evidence for the previously proposed wave of interdependent motions of TM helices (57) within the Orai channel upon pore opening. This assumption of a global concerted gating motion is based on the recent discovery of a series of GoF mutants throughout all TM domains (57). Our library of GoF–LoF double point mutants (Table 1) unveils a dominant inhibitory effect of a series of LoF over most GoF mutations independent of their distance to the pore and their location relative to each other (Table 1). This suggests that global instead of local conformational changes within the entire Orai complex are fundamental for pore opening. We discovered that these gating motions are controlled by crucial checkpoints, located predominantly in the conical MTR-ring, the CETR including the cytosolic triangles. LoF mutations at these key positions interfere mainly with an open pore geometry. As long as clearance of all these critical checkpoints is achieved, pore opening of the CRAC channel complex occurs. On the contrary, a single position in a nonpermissive state inhibits Orai channel opening. Thus, these complex correlations can be best described by the principles of Boolean algebra. We show that the Orai pore opening mechanism in dependence of gating checkpoints in the MTR and CETR follows the rules of the truth table of a logical “AND” gate as shown in Figures 8*A* and S9.

A significant feature of several residues in the conical MTR ring is that their single point mutation can lead either to gain (MTR-GoF) or loss of function (MTR-LoF). Thus, these residues possess two roles: (1) maintaining the closed state of the Orai channel, as mutations can lead to constitutive activation likely owing to the release of steric brakes as recently studied in detail for the position H134 (51, 57) and (2) contributing to the establishment of an opening-permissive pore and channel conformation, as other mutations can abolish function likely owing to a dewetting of the hydrophobic gate.

Critical checkpoints located within the CETR, in particular in the cytosolic triangles and the hydrophobic modules, establish an opening-permissive pore geometry as revealed by cysteine cross-linking and MD simulation studies. Within the cytosolic triangles of two adjacent subunits, two intra-Orai1 and one inter-Orai1 salt-bridge interactions formed by basic and acidic residues in TM1, TM2, and TM3 are relevant in establishing an opening-permissive pore conformation. Although the available closed and open Orai structures indicate that two of them (K85–E173, K85–E149) are already in close proximity, one (R83–E149) seems to require larger rearrangements to form a stable salt bridge. K85 in Orai1 appears to be a critical pivot for the channel through its interactions with E149 and E173. These triangular interactions possess probably two major functions: (1) they prevent the pore from collapsing and (2) they keep E149 and E173 close to each other in preparation for further conformational reorientations during pore opening after STIM1 binding. The latter refers, for instance, to the functional importance of the R83–E149 salt bridge (Fig. 8*B*). We suppose that it is preferentially formed in the presence of STIM1. In line with this hypothesis, Dong *et al*. (60) suggested that upon channel opening rotation in TM1 might happen. Then, R83 can get in contact with E149, which is kept in the vicinity owing to its interaction with K85 allowing the pore to further expand. This new interaction seems to act as a second lock, similar to K85–E173, to keep the channel in a fully open state. Moreover, we assume that it assists to stretch the hydrophobic gate even further to increase the level of hydration, which facilitates the permeation of Ca^2+^ ions through the channel. Our functional studies further elucidated that at least two of these three salt bridges are required to be intact for a functional pore conformation and the maintenance of STIM1-mediated Orai1 activation (Fig. 8*B*). Of interest, these correlations can be illustrated by the logical operators for the AND and OR gates as shown in Figure S9.

Our pool of double point mutants containing one MTR-GoF and one MTR- or CETR-LoF in a variety of combinations provide one key step further in the detailed understanding of the motions within the Orai complex upon pore opening. A diversity of LoF mutations prevent a GoF mutant to switch into the open state, likely *via* the introduction of inter-TM constraints. Thus, we propose that the Orai1 activation signal, induced by STIM1 coupling to the Orai1 C terminus, propagates like a spherical wave of inter-dependent TM motions within one subunit to the entire hexameric channel complex (Fig. 8*C*) to induce pore opening. This concept is especially supported by three observations: (1) Double mutants containing a GoF and a LoF point mutation, independent of which one located in the 10.13039/501100010693TM domain closer to the pore, loses function. (2) LoF mutations are dominant over GoF mutations not only within the same (*e.g.*, MTR-LoF and MTR-GoF) but also over distinct membrane planes (*e.g.*, CETR-LoF over MTR-GoF such as L174D over H134A; Fig. 8*C*; Table 1). (3) The dominant inhibitory effect holds also for some combinations of LoF (E149K, L174D) and GoF mutations, when each is located within a distinct subunit of a dimer. Concerning the latter, the requirement of more than three intact Orai subunits is in line with the nonlinear dependence of Orai activation and STIM1 coupling (69). The transmission of the activation signal across the entire Orai channel complex is likely enforced by the TM2/3 ring, which has recently been proposed to function as an individual unit that devolves strong cooperativity between STIM1 coupling at six single subunits and pore opening (57, 70, 71).

Among the constitutively activating Orai1 point mutations, the V102A in TM1 is the only GoF mutation that acts dominantly over MTR- and CETR-LoF point mutations. These findings suggest that the extent of pore hydration of Orai1 V102A (59) is sufficient to confer Ca^2+^ permeation. This exceptional behavior of Orai1 V102A TM1 pore mutant is also accompanied by a number of distinctly different biophysical hallmarks distinguishing it from the TM2-TM4- constitutive point mutants. Only Orai1 V102A leads to a strong reduction of V_rev_ and an enhancement of I_DVF_ versus I_Ca2+_, allows the permeation of Cs^+^ ions, and maintains the activity upon N-terminal deletions (37, 41, 51). Thus, Orai1 V102A activation occurs *via* pronounced pore dilation or distinct interdependent motions of the TM helices, which is likely responsible for its unique biophysical characteristics (41) that are so different from other Orai-activating mutants.

Whether the global conformational changes within the Orai complex upon pore opening are provoked *via* the STIM1 coupling to Orai1 C terminus or additional other cytosolic domains is still unclear. At present, one hypothesis is that STIM1 coupling to the Orai1 C terminus is sufficient to trigger the allosteric movements within the channel complexes (54). An alternative hypothesis is that the interdependent TM movements are additionally triggered by STIM1 coupling to the loop2 and the N-terminal segments (39). The effect of LoF mutations enabled us to elucidate sites critical for STIM1 coupling and to clarify their significance relative to each other. Only the inhibitory effect of MTR-LoF and the CETR- K85E mutations can be partially overcome by coexpression of OASF L251S with Orai1 or by the local attachment of CAD fragments direct onto the respective Orai protein. Moreover, dimers containing one subunit with a GoF mutation and the second subunit with either an MTR-LoF or the CETR-K85E mutation remain functional. This indicates that an opening-permissive pore geometry is retained or at least can be forced by an adequate amount of STIM1 bound to Orai channels. In contrast, other CETR-LoF mutations (*e.g.*, E149, L174) prevent these alternative activation pathways. In line with these findings, colocalization studies revealed that the MTR-LoF and the CETR-LoF Orai1 K85E mutations affect STIM1 coupling only mildly, whereas other CETR-LoF mutations impede STIM1 coupling. This behavior is also reflected by the V_rev_ of the respective LoF mutants in the V102A background, thus functioning as a readout parameter for STIM1 coupling (35, 37). Hence, gating checkpoints in or close to the loop2 region seem to contribute to the formation of an STIM1 coupling site, either directly or indirectly. Accordingly, the loop2 segment has been suggested to be involved in STIM1-mediated gating (40). Moreover, the N terminus together with the loop2 region is required to establish an opening-permissive pore conformation and to fine-tune authentic CRAC channel hallmarks together with STIM1 (39). Thus, the loop2 with E149 and L174 is beside the Orai1 C terminus, the second indispensable prerequisite for a fully intact STIM1–Orai1 coupling. Owing to the more potent inhibitory role of E149K and L174D compared with K85E, it is tempting to speculate that the loop2 region functions as an essential bridge for transmission of the Orai1 activation signal from the Orai1 C terminus to the pore.

In addition to the indispensable checkpoints in the MTR and CETR, the MTR harbors several positions only crucial for STIM1-mediated activation but not for H134A-induced activation and STIM1 binding as determined in Figures 4*A* and S4, *A–D*. This suggests partly distinct requirements in the signal propagation pathways of STIM1- and H134A-mediated activation. Moreover, the inhibitory action to STIM1 coupling and -mediated activation of LoF mutations in the hinge region (3xA, 3xG, L261D) can be bypassed by MTR-GoF mutations (H134A, P245L). Thus, we assign to the hinge region a potent role in STIM1 coupling in line with Zhou *et al*. (54), besides its potential effect on the pore architecture.

Overall, we prove that Orai1 pore opening comes along with and indispensably requires opening-permissive, interdependent TM domain motions across the entire channel complex. This requires clearance of a number of gating checkpoints in the middle and cytosolic segments of the Orai TM domains. A core element for STIM1 coupling and Orai1 pore opening is established by cytosolic triangular salt-bridge interactions within one and between neighboring Orai1 subunits. A synergistic action of these gating checkpoints together with STIM1 coupling triggers pore opening and ensures high Ca^2+^ selectivity. The variety of elucidated mutants represent promising tools in future drug development to interfere with one of the multiple gating checkpoints to manipulate Orai1 function. Moreover, they provide a valuable source for novel structural insights.

## Experimental procedures

### Molecular biology

For N-terminal fluorescence labeling of human Orai1 (Orai1; Accession number NM_032790_3, provided by A. Rao’s lab) the constructs were cloned into the pEYFP-C1 (Clontech) expression vector *via* KpnI and XbaI (Orai1) restriction sites. Site-directed mutagenesis of all the mutants was performed using the QuikChange XL site-directed mutagenesis kit (Stratagene) with the corresponding Orai1 constructs serving as a template.

Human STIM1 (STIM1; Accession number: NM_003156) N-terminally ECFP tagged was kindly provided by T. Meyer’s Lab, Stanford University. pECFP-C1 STIM1 C terminus (aa233-685 wt and L251S) was used as a template for the generation of pECFP-OASF (wt and L251S) by introducing a stop codon at position 475 (aa 233–474) using the QuikChange XL site-directed mutagenesis kit (Stratagene). STIM1 fragment 344 to 449 (CAD) was amplified *via* PCR including an N-terminal KpnI and a C-terminal XbaI restriction site for cloning into the pECFP-C1 vector.

### Orai1 dimers with a linker

Point mutations (H134A/S239W) were introduced into peYFP-C1/Orai1 (peYFP-C1, Clontech) plasmids. YFP-Orai1 dimers were cloned using suitable restriction sites. Linker regions (RDPLVQGGGSGGCGGIAL) between Orai1 cDNAs were constructed using QuikChange site-directed mutagenesis kit (Agilent Technologies) by the use of suitable primers inserting amino acids GGGSGG in dimers that included the short linker region (RDPLVQ∗CGGIAL).

### Orai1-CAD-CAD constructs

The Orai1-CAD-CAD-GFP constructs were designed by cloning Orai1 and STIM1-CAD domains (aa344–449) into peYFP-N1 (Clontech). The linker regions between the Orai1 and CAD (Orai1-linker-1-CAD-linker-2-CAD-YFP) domains were constructed using QuikChange site-directed mutagenesis with suitable primers (linker-1: SSRAGGGGSGGGGS; linker-2 GGSGGGSSPRGGGGGSGGGGS) inserting desired amino acids.

The integrity of all resulting clones was confirmed by sequence analysis (Eurofins Genomics/Microsynth).

### Cell culture and transfection

The transient transfection of HEK293 cells was performed (72) using the TransFectin Lipid Reagent (Bio-Rad) (New England Biolabs). Regularly, potential cell contamination with mycoplasma species was tested using VenorGeM Advanced Mycoplasma Detection kit (VenorGeM).

### Cell preparation, cysteine cross-linking, and Western blot analysis

HEK293T cells cultured in 12-cm dishes were transfected with 5 μg plasmid using Transfectin lipid reagent (Bio-Rad) following the manufacturer’s instructions. Twenty-four hours after transfection, cells were harvested and washed twice in a Hank’s balanced salt solution buffer containing 1 mM EDTA. After centrifugation (1000*g*/2 min), cell pellets were resuspended in homogenization buffer (25 mM Tris HCl pH 7.4, 50 mM NaCl, protease inhibitor [Roche]) and incubated on ice for 15 min. The lysed cells were passed 10 times through a 27G ½” needle and centrifuged at 1000*g* for 15 min at 4 °C to pellet debris. The cell lysates were treated with 1 mM CuP-solution for 5 min at room temperature, and the reaction was stopped upon addition of 50 mM N-ethylmaleimide quenching solution. A total of 21 μl of each sample was mixed with nonreducing Laemmli’s buffer, heated 15 min at 55 °C, and subjected to a 12% SDS-PAGE. Separated proteins were transferred to a nitrocellulose membrane and immunoblotted with an antibody recognizing Orai1 (Sigma-Aldrich, cat. No. O8264 and a corresponding horseradish peroxidase–conjugated anti-rabbit antibody). Each experiment was performed at least three independent times and analyzed with the program ImageJ (NIH) to calculate the percentage of dimer formation.

### Electrophysiology

Electrophysiological recordings that assessed the characteristics of 2 to 3 constructs were carried out in paired comparison on the same day. Expression patterns and levels of the various constructs were carefully monitored by confocal fluorescence microscopy and were not significantly changed by the introduced mutations. Electrophysiological experiments were performed at 20 to 24 °C, using the patch-clamp technique in the whole-cell recording configuration. For STIM1/Orai as well as STIM1 C terminus/Orai current measurements, voltage ramps were usually applied every 5 s from a holding potential of 0 mV, covering a range of –90 to +90 mV over 1 s. The internal pipette solution for passive store depletion contained (in mM) 3.5 MgCl_2_, 145 Caesium Methane Sulphonate, 8 NaCl, 10 Hepes, 20 EGTA, pH 7.2. The extracellular solution consisted of (in mM) 145 NaCl, 5 CsCl, 1 MgCl_2_, 10 Hepes, 10 glucose, 10 CaCl_2_, pH 7.4. The applied voltages were not corrected for the liquid junction potential, which was determined as +12 mV. All currents were leak corrected by subtraction of the leak current, which remained following 10 μM La^3+^ application.

Bar graphs in the figures display for Orai1 proteins in the absence of STIM1 the current density at t = 0 s, while in the presence of STIM1 maximum current densities are shown.

### Confocal fluorescence microscopy

Confocal microscopy for colocalization experiments was performed similarly to (73). In brief, a QLC100 Real-Time Confocal System (VisiTech Int, UK) was used for recording fluorescence images connected to two Photometrics CoolSNAPHQ monochrome cameras (Roper Scientific, USA) and a dual port adapter (dichroic: 505lp; cyan emission filter: 485/30; yellow emission filter: 535/50; Chroma TechnologyCorp, USA). This system was attached to an Axiovert 200M microscope (Zeiss, Germany) in conjunction with two diode lasers (445 and 515 nm) (Visitron Systems). Image acquisition and control of the confocal system was performed with a Visiview 2.1.1 software (Visitron Systems). Illumination times for CFP and YFP images that were consecutively recorded with a minimum delay were about 900 ms.

### Molecular dynamic simulation protocols

A homology model of hOrai1 based on the crystal structure (Protein Data Bank ID: 4HKR) (42) was used through all the simulations following the procedure described in Frischauf *et al.* (63). Both the wildtype and Orai mutants were embedded in a pure palmitoyl-2-oleoyl-sn-glycerol-3-phospho**c**holine or also known as 1,2-palmitoyl-oleoyl-*sn*-glycero-3-phosphocholine membrane composed by 388 lipids using the CHARMM-GUI web interface (74). The TIP3p (75) model was used to represent water molecules. The CHARMM36 forcefield was used to treat proteins (76) and lipids (77). Calcium and chloride ions were used to neutralize the system with an ionic strength set at 100 mM. One calcium ion was placed in the selectivity filter according to the crystal structure before running any simulations. Ions were treated by using a rescaled charge paradigm (78, 79, 80, 81). These parameters have been shown to better reproduce the properties of the ion in water and its hydrated structure (81). Interactions with the backbones of proteins and the free energy of binding of Ca^2+^ ions to Ca^2+^-signaling proteins (82) are also improved.

Mutations were performed using the following protocol. A wildtype Orai configuration was extracted from a 200-ns-long simulation. The *in silico* mutation tool of the modeling program Yasara (83) was used to introduce the mutations.

The GROMACS (84) software version 5.1.4 was used to perform all MD simulations. The temperature was set at 310K under a pressure of 1 atm. Minimization and equilibration followed the six-step CHARMM-GUI protocols (85, 86). The 250- and 400-ns-long molecular dynamics production simulations were performed for single and double point mutants, respectively.

VMD (87) was used to visualize the simulations. VMD and MD analyses (88) were used to analyze the simulations. Figures were generated using Matplotlib (89). The last 50 ns of each trajectory were used for analysis.

### Statistics

Results are presented as scatter blots means ± SD (in block diagram except Fig. S1) or SEM (in time courses) calculated for the indicated number of experiments. The Mann–Whitney test was performed for statistical comparison of two independent samples considering differences statistically significant at *p* < 0.05. In case of variance homogeneity (determined by the Levene test), the ANOVA test was used for statistical comparison of multiple independent samples using the F-distribution. In cases where variance homogeneity was not fulfilled we performed instead of the ANOVA test, the Welch–ANOVA test. Subsequent to the ANOVA test, we performed the Fisher least significant difference post hoc test to determine the pairs that differ statistically significantly (*p* < 0.05). Subsequent to Welch–ANOVA we performed the Games–Howell post hoc test to determine the pairs that differ statistically significantly (*p* < 0.05). The Shapiro–Wilk test was applied to prove normal distribution of the respective datasets.

## Data availability

All data are contained within the article.

## Conflict of interest

The authors declare that they have no conflicts of interest with the contents of this article.
